# P4HA2 hydroxylates SUFU to regulate the paracrine Hedgehog signaling and promote B-cell lymphoma progression

**DOI:** 10.1038/s41375-024-02313-8

**Published:** 2024-06-22

**Authors:** Quanfu Li, Yiyang Liu, Jingxian Wu, Zewen Zhu, Jianjun Fan, Linhui Zhai, Ziruoyu Wang, Guiping Du, Ling Zhang, Junchi Hu, Dengke K. Ma, Jun O. Liu, Hai Huang, Minjia Tan, Yongjun Dang, Wei Jiang

**Affiliations:** 1 Key Laboratory of Metabolism and Molecular Medicine, Ministry of Education, Department of Biochemistry and Molecular Biology, School of Basic Medical Sciences, Shanghai Medical College, Fudan University, Shanghai, 200032 China; 2 Department of pathology, College of Basic Medicine, Molecular Medicine Diagnostic and Testing Center, Department of Pathology, the First Affiliated Hospital of Chongqing Medical University, Chongqing Medical University, Chongqing, 400016 China; 3 Basic Medicine Research and Innovation Center for Novel Target and Therapeutic Intervention, Ministry of Education, College of Pharmacy, Chongqing Medical University, Chongqing, 400016 China; 4 Translational Research Institute of Brain and Brain-Like Intelligence, Shanghai Fourth People’s Hospital, School of Medicine, Tongji University, Shanghai, 200434 China; 5 Women’s Hospital, Zhejiang University School of Medicine, Hangzhou, 310058 China; 6 Basic Medicine Research and Innovation Center for Novel Target and Therapeutic Intervention, Ministry of Education, The Second Affiliated Hospital of Chongqing Medical University, Chongqing, 400016 China; 7 Department of Physiology, Cardiovascular Research Institute, University of California San Francisco, San Francisco, CA 94158 USA; 8 Department of Pharmacology and Molecular Sciences, The Johns Hopkins University School of Medicine, Baltimore, MD 21287 USA; 9 Department of Cell Biology, and Second Affiliated Hospital, Zhejiang University School of Medicine, Hangzhou, 310058 China; 10 State Key Laboratory of Drug Research, Shanghai Institute of Materia Medica, Chinese Academy of Sciences, Shanghai, 201203 China

**Keywords:** Oncogenes, Biochemistry

## Abstract

Aberrations in the Hedgehog (Hh) signaling pathway are significantly prevailed in various cancers, including B-cell lymphoma. A critical facet of Hh signal transduction involves the dynamic regulation of the suppressor of fused homolog (SUFU)-glioma-associated oncogene homolog (GLI) complex within the kinesin family member 7 (KIF7)-supported ciliary tip compartment. However, the specific post-translational modifications of SUFU-GLI complex within this context have remained largely unexplored. Our study reveals a novel regulatory mechanism involving prolyl 4-hydroxylase 2 (P4HA2), which forms a complex with KIF7 and is essential for signal transduction of Hh pathway. We demonstrate that, upon Hh pathway activation, P4HA2 relocates alongside KIF7 to the ciliary tip. Here, it hydroxylates SUFU to inhibit its function, thus amplifying the Hh signaling. Moreover, the absence of P4HA2 significantly impedes B lymphoma progression. This effect can be attributed to the suppression of Hh signaling in stromal fibroblasts, resulting in decreased growth factors essential for malignant proliferation of B lymphoma cells. Our findings highlight the role of P4HA2-mediated hydroxylation in modulating Hh signaling and propose a novel stromal-targeted therapeutic strategy for B-cell lymphoma.

## Introduction

The Hh signaling pathway plays a pivotal role in fundamental biological processes, governing embryonic development and adult tissue homeostasis. Dysregulation of this pathway is implicated in diverse cancers, including B-cell lymphoma, leading to neoplastic transformations, malignancies, and drug resistance [1]. Initiated by the binding of Hh ligand to its receptor Patched1 (PTCH1), this pathway activation involves the dissociation of PTCH1 from Smoothened (SMO), culminating in the activation of GLI transcription factors and subsequent transcription of downstream target genes.

SUFU acts as a dominant suppressor within the Hh pathway, forming a complex with GLIs to restrain their transcriptional activities [2, 3]. Primary cilia, maintained by KIF7, play a crucial role in Hh signal transduction [4, 5]. KIF7 localizes to the distal tip of primary cilium, creating a compartment pivotal for the propagation of Hh signaling. Upon activation, core pathway components, including the SUFU-GLI complex, translocate to the distal cilia tips, where the complex dissociates, allowing GLIs enter the nucleus and initiate downstream genes transcription [4, 6–8]. Protein post-translational modifications, such as phosphorylation of GLI and SUFU, have critical roles in dynamic Hh pathway regulation [9–22]. Despite diverse roles, the mechanism underlying the dissociation of the SUFU-GLIs complex remains unexplored.

Deregulation of any component within the Hh pathway leads to malignant tumor progression, making it a prominent therapeutic target in oncology. Mechanisms of aberrant Hh signaling include ligand-independent constitutive activation, ligand-dependent autocrine signaling, and ligand-dependent paracrine signaling [23]. The third mechanism involves ligand-dependent paracrine signaling transduction, frequently utilized in tumorigenesis for intercellular communication. Tumor cells secrete the Hh ligands, activating the signaling cascade in surrounding stromal cells. In turn, these stromal cells produce factors that support and promote tumor cell transformation in a feedback loop [24]. Additionally, tumor cells receive Hh signals from bone marrow or lymph node stromal cells, promoting their own proliferation, a phenomenon reported mainly in hematological malignancies such as B-cell lymphoma [25]. However, the activation of the Hh pathway in stromal cells in this manner has not been fully clarified.

Protein proline hydroxylation, catalyzed by prolyl 4-hydroxylases, represents another layer post-translational modification. These enzymes are classified into two families based on their substrates. The prolyl hydroxylase domain-containing protein (PHD) family is responsible for hydroxylating and regulating the degradation of the key factors in various cellular processes, including hypoxia-inducing factor 1α (HIF1α) [26–28], AKT [29], P53 [30], FOXO3a [31], and other substrates [32–35]. The other family is the collagen prolyl 4-hydroxylase family (C-P4H), consisting of three main isoforms (P4HA1, P4HA2, and P4HA3), crucial for maintaining the thermal stability of the collagen triple helix. Unlike PHDs, the substrates and biological functions of C-P4Hs are still largely unknown beyond collagen. Some proteins with collagen-like sequence, such as apoproteins [36] and Argonaute 2 [37], have been identified as C-P4Hs substrates. C-P4Hs are reported to be highly expressed in various tumors, including breast cancer, hepatocellular carcinoma, lung cancer, glioma, and prostate cancer, mainly influencing collagen synthesis and thereby contributing to tumor growth and metastasis. Furthermore, C-P4Hs have been reported proposed as potential targets for tumor intervention.

In our previous work, we identified P4HA2 as a key player in the hydroxylation of Carabin, a new substate exclusively expressed in lymphocytes. This modification, occurring at proline 306, facilitates the degradation of Carabin and activities ERK pathway, thereby promoting B-cell lymphoma progression [38]. Notably, within diffuse large B-cell lymphoma (DLBCL) tissues, P4HA2 is expressed in tumor cells in approximately half of the tumor samples, while exhibiting heightened expression in stromal cells surrounding tumor in the majority of tumor samples. Despite these observations, the specific role of stromal P4HA2 in the progression of B-cell lymphoma remains ambiguous and warrants further investigation.

To further unravel the function of P4HA2, we embarked on identifying its interacting proteins using P4HA2 as a bait. In this pursuit, we discovered KIF7, a crucial component of the Hh pathway, as a major binding partner of P4HA2. Subsequent investigations unveiled that upon activation of the Hh signaling, KIF7 co-migrates with P4HA2 from the cytoplasm to the tip of cilium. Notably, we found that P4HA2 plays a role in the hydroxylation of SUFU, a dominant suppressor in the Hh pathway, ultimately activating the Hh signaling. We constructed a B-cell lymphoma mouse model to demonstrate that P4HA2 is expressed in stromal cells to promote B-cell lymphoma progression via regulating the Hh signaling. Collectively, our observations suggest that P4HA2, along with the proline hydroxylation of SUFU, plays crucial roles in promoting B-cell lymphoma progression through a paracrine signaling transduction mechanism.

## Methods

### Antibodies and reagents

The following antibodies were used for western blotting (WB), immunofluorescence (IF): PAN-OH (home-made; 1:1,000 for WB); P4HA2 (13759-1-AP, rabbit, Proteintech; 1:1,000 for WB, 1:100 for IHC, 1:200 for IF); P4HA1 (12658-1-AP, rabbit, Proteintech; 1:1,000 for WB); KIF7 (24693-1-AP, rabbit, Proteintech; 1:1,000 for WB, 1:100 for IHC); SUFU (26759-1-AP, rabbit, Proteintech; 1:2,000 for WB); Flag (clone M2, F1804, mouse, Sigma-Aldrich; 1:10,000 for WB, 1:200 for IF); His (clone OGHis, D291-3, mouse, Medical & Biological Laboratories; 1:5,000 for WB); V5 (AB3792, rabbit, Merck Millipore; 1:2,000 for WB); GLI1 (clone A-7, sc-515781, mouse, Santa Cruz; 1:1,000 for WB, 1:200 for IHC); GLI2 (18989-1-AP, rabbit, Proteintech; 1:1,000 for WB, 1:100 for IHC) ; GAPDH (clone 1E6D9, 60004-1-Ig, mouse, Proteintech; 1:10,000 for WB); Arl13b (clone N295B/66, 75-287, mouse, NeuroMab; 1:200 for IF); Cy3 AffiniPure Goat Anti-Mouse IgG (H + L) (115-165-003, Jackson ImmunoResearch; 1:10,000 for IF); α-SMA (#34105, Cell Signaling Technology, 1:100 for IF) Light chain specific Mouse Anti-Rabbit IgG (211-032-171, Jackson ImmunoResearch; 1:10,000 for WB); Light chain specific Goat Anti Mouse IgG (115-035-174, Jackson ImmunoResearch; 1:10,000 for WB); Peroxidase AffiniPure Goat Anti-Rabbit IgG, Fc fragment specific (111-035-008, Jackson ImmunoResearch; 1:10,000 for WB); Donkey anti-mouse IgG (H&L) (715-035-150, Jackson ImmunoResearch; 1:10,000 for WB); Goat Anti-Rabbit IgG (H + L) (111-035-003, Jackson ImmunoResearch; 1:10,000 for WB); Peroxidase AffiniPure Goat Anti-Mouse IgG, Fcγ fragment specific (115-035-008, Jackson ImmunoResearch; 1:10,000 for WB); Smoothened Agonist (SAG), Selleck, Cat# S7779; Fetal bovine serum, Thermo Fisher, Cat# 16140071; Penicillin/streptomycin, Thermo Fisher, Cat# 15140122; RPMI 1640, Thermo Fisher, Cat# 11875093; DMEM, Thermo Fisher, Cat# 11995065; Puromycin Dihydrochloride, Sigma, Cat# P8833; Trypsin 0.25% EDTA, Thermo Fisher, Cat# 25200072; Polyethylenimine Hydrochloride (MW 40,000), Polysciences, Cat# 24765-1; Polybrene Transfection Reagent, Sigma, Cat# TR-1003-G; SHH protein, Yun Zhao Laboratory; Anti-V5-tag mAb-Magnetic Beads, Medical & Biological Laboratories, Cat# M215-11; NTI-FLAG® M2 Affinity Gel, Sigma, Cat# F2426; Ni-NTA Agarose, QIAGEN, Cat# 30230; HiScript II 1st Strand cDNA Synthesis Kit (+gDNA wiper), Vazyme, Cat# R212-01/02; ChamQ SYBR qPCR Master Mix, Vazyme, Cat# Q311-02.

### Tandem affinity purification

The P4HA2 gene was cloned into a pcDNA3.1 plasmid which coding protein A and streptavidin binding protein in its N terminal. 2 × 10^7^ HEK293T cells were seeded in 15 cm dish. 20 μg plasmids was transfected with 60 μL PEI in DMEM with 10% FBS without PS next day. One day after transfection, the cells were harvest with lysis buffer containing 50 mM Tris-HCl (pH 7.5), 125 mM NaCl, 0.2% NP-40, 5% glycerol, 1.5 mM MgCl_2_ and protease inhibitors (Sigma) and phosphatase inhibitor cocktail (Sigma) and lysate with sonicate. Then the supernatant was incubated with 30 μL IgG beads (Sigma) for 2 hr at 4 °C, IgG beads were then incubated with 1.5 μL AcTEV protease (Invitrogen, 10 U/μL) per tube at 4 °C overnight. 80 μL Streptavidin beads (Invitrogen) per tube was equilibrated with SBP buffer containing 10 mM Tris-HCl (pH 7.5), 100 mM NaCl and 0.2% NP-40. IgG beads treated with the AcTEV was spun down and the supernatant was added to the streptavidin beads and incubate at 4 °C for 4 hr. Streptavidin beads was finally spined down and boiled with 40 μL 2× SDS sample buffer and taken to the Mass spectrum analysis.

### Pulldown and co-immunoprecipitation

HEK293T cells transfected with plasmids were pelleted by centrifugation, washed with 1X PBS and lysed for 15 min in RIPA buffer at 4 °C (50 mM Tris-HCl pH 8, 150 mM NaCl, 1% NP-40, 0.5% sodium deoxycholate, 0.1% SDS) or Cell Lysis Buffer (Cell Signaling) supplemented with protease inhibitors (Sigma) and phosphatase inhibitor cocktail (Sigma). Following centrifugation at 12,000 rpm at 4 °C for 15 min, supernatants were recovered. 10% volume of whole cell lysates were collected as input. Lysates were incubated with 30 μL Ni NTA agarose (QIAGEN) or Flag beads (Sigma) at 4 °C for 3 hr, and then the beads were washed four times with lysis buffer and boiled with sample buffer, then separated on 10% SDS-PAGE gel together with 0.1% input.

### Cell culture

NIH/3T3(a gift from Ma Dengke at University of California, San Francisco) or HEK293T(a gift from Ma Dengke at University of California, San Francisco) cells were cultured in DMEM (Thermo Fisher) supplemented with 10% fetal bovine serum (FBS) and 1% penicillin/streptomycin/glutamine (Thermo Fisher) in a humidified 5% CO_2_ incubator at 37 °C. OP9 cells (obtained from National Collection of Authenticated Cell Cultures, China) were cultured in MEM-alpha (Thermo Fisher) with 10% fetal FBS 1% penicillin/streptomycin/glutamine (Thermo Fisher) in a humidified 5% CO_2_ incubator at 37 °C. *Eµ-myc arf*^*−/*^^−^ B cells (a gift from Jiang Hai at Chinese Academy of Sciences [39]) were cultured in DMEM and IMDM (Thermo Fisher) supplemented with 10% fetal bovine serum (FBS), 1% 50 mM beta-ME and 1% penicillin/streptomycin/glutamine (Thermo Fisher) in a humidified 5% CO_2_ incubator at 37 °C.

All cell lines were free of mycoplasma contamination. Experiments assaying the Hh signaling were carried out in 0.5% FBS supplemented with Smoothened Agonist (SAG) (Sigma). Mammalian cell transfection performed using Polyethylenimine Hydrochloride (MW 40,000) Transfection Reagent (Polysciences) manufacturer protocol.

### Co-culture and cell viability assays

OP9 and mice primary bone marrow cells were plated in 6-well plates as required and allowed to attach and grow for 48 hr. For co-culture, cell culture supernatant was collected and *Eµ-myc arf*^*−/*^^−^ B cells were cultured in the supernatant. Cell viability assay was done using CellTiter-Glo® (Promega).

### The Hedgehog signaling transduction

NIH/3T3, HEK293T or OP9 cells were cultured for over 12 hr, then 10% FBS was replaced by 0.5% FBS overnight. SAG (200 nM) or the biological ligand, Sonic Hedgehog (Human), recombinant protein (200 ng/mL) treated cells for 24 hr. The Hh pathway targeted genes were analyzed by qRT-PCR. Primers sequences for qRT-PCR are shown in Supplementary Table 2.

### The Hedgehog reporter activity assay

NIH/3T3 cell line stably transfected with Gli-dependent firefly luciferase and constitutive *Renilla* luciferase reporters [40], which was cultured for over 12 hr, then 10% FBS DMEM was replaced by 0.5% FBS DMEM overnight. SAG (200 nM) treated cells for 24 hr. Lyse cells with 1x whole cell lysis buffer available from the Dual-Luciferase Reporter Assay System and collect aliquot of supernatant (20 μL) from cell lysis, plate them into 96-well plate. Thaw dual-luciferase reporter reagents. Flash and prime Berthold luminometer with firefly luciferase and *Renilla* luciferase substrate reagents. 20 μL of each substrate is added sequentially by the luminometer and light signals generated are instantly measured by the EnSpire. Read firefly and *Renilla* luciferase signals (firefly luciferase signal is detected at 560 nm and *Renilla* luciferase signal is detected at 480 nm).

### Fluorescence microscopy

NIH/3T3 were co-infected with indicated viruses, which overexpressed KIF7-BFP, P4HA2-EGFP and Arl13b-mCherry for 48 hr. Puromycin was used to select the positive cells and to maintain the pressure of selection. Cells were seeded on the 35-mm glass bottom dish overnight, then cells were serum starved for 12 hr and then treated with SAG (200 nM) for 24 hr. Imaging was done with a ZEISS LSM 980 with Airyscan 2 microscope equipped with a C-plan-Apochromat 63x/1.4 objective. The excitation and emission spectra of BFP, GFP and mCherry was 405 nm 1.5%/574-720 nm, 488 nm 6.5%/420-480, 495-550 nm, 561 nm 2.6% / 300-720 nm respectively.

### Cell Immunofluorescence staining

NIH/3T3 were co-infected with indicated viruses, which overexpressed KIF7-BFP, P4HA2-EGFP for 48 hr. Puromycin was used to select the positive cells and to maintain the pressure of selection. Cells were seeded on the glass coverslips in 24-well plate overnight, then cells were serum starved for 12 hr and then treated with SAG (200 nM) for 24 hr. Cells were fixed with 4% paraformaldehyde in PBS buffer, and were incubated for 15 min at room temperature. Then, samples were blocked by incubation in 5% normal horse serum in PBS. Primary cilia were detected by Arl13b (1:200, NeuroMab), and the second antibody was Cyanine 3 (1:2,000, Jackson ImmunoResearch). Samples were mounted in polyvinyl-based mounting media (Southern Biotech), and were imaged with A1R Multiphoton laser confocal microscope (Nikon).

### Lentiviral transduction

Knockout or knockdown: CRISPR/Cas9 genome editing was used to generate stable knockout cell lines. Cells were co-transfected with lentiCRISPR v2-EGFP (Knockout) or lentiviral shRNA (knockdown) expression vector, the lentiviral packaging vectors psPAX2 and the vesicular stomatitis virus G glycoprotein expression vector pMD2.G using PEI reagent. The supernatant containing lentivirus was collected at the second and third day after transfection, followed by filtration (Acrodisc Syringe Filter 0.45 μm Supor Membrane, PALL Life Sciences) and concentration (Millipore 100 μM). Viral supernatant was used to infect 1 × 10^5^ cells in a 35-mm dish with 5 μg/mL polybrene (Sigma), followed by 1500 rpm centrifugation for 2 hr at room temperature. The Knockout cell lines were determined by sequencing and Western blotting. The knockdown efficiency was determined by Western blotting. The sgRNA or shRNA sequences are shown in Supplementary Table 2.

Overexpression: coding sequences of P4HA2, KIF7 and GLI1 were fused with GFP, BFP and V5 tag and cloned into lentiviral constructs, respectively. Lentiviral constructs were packaged in HEK293T and transduced with NIH/3T3. Stably positive colonies were sorted by puromycin and expanded. The overexpression efficiency was determined by Western blotting.

### In-gel digestion and LC-MS/MS analysis

The protein was analyzed by SDS-PAGE and the aim gel bands were cut. The in-gel digestion was according to the published methods [41]. Briefly, the gel bands were cut into 1 mm^3^ particles. The protein-cysteine residues were reduced by adding 5 mM dithiothreitol buffer at 56 °C for 1 hr. Then the cysteine residues were alkylated with 10 mM iodoacetamide in the dark at room temperature for 30 min. Proteins were digested by sequence grade trypsin enzyme at a trypsin- to-protein ratio of 1:50 (w/w). The trypsin digestion was incubated at 37 °C for 16 hr. Peptides were extracted and desalted by C_18_ Zip-Tips. The desalted peptides were stored in -80 °C before LC-MS/MS analysis.

The dried peptides were dissolved in buffer A (0.1% formic acid in 2% acetonitrile) and then were loaded onto the home-made capillary C_18_ column (75 µm ID x 20 cm length, packed with C_18_ resins, 3 µm particle size, 100 Å pore size (Dikma Technologies) equipping on Easy-nanoLC 1000 system (Thermo Scientific). The peptides were eluted from column by 60 min gradient at flow rate of 350 nL/min. The gradient used was as follows: 5 – 32% buffer B (0.1% formic acid in 90% acetonitrile) over 40 min, 32 – 45% buffer B over 15 min, 45 – 80% buffer B over 2 min and then kept at 80% for 3 min. The eluted peptides were ionized under high voltage (2.1 kV) and detected by Orbitrap Fusion mass spectrometer (Thermo Scientific). For the full scan, the ions were detected by Orbitrap analyzer with mass range of 350-1550 m/z at a resolution of 120,000 (m/z 200). The Automatic Gain Control (AGC) targets were set at 5 × 10^5^ and the maximum injection time was set as 50 milliseconds (msec). The MS/MS acquisition was used by top speed mode and the cycle time was set as 3 s (sec). The precursor ions were fragmented under higher energy collision dissociation (HCD) with normalized collision energy (NCE) of 32%. The fragment ions were detected by ion trap. The AGC for the MS/MS were set to 7 × 10^3^. The maximum injection time was 50 msec and dynamic exclusion was 30 sec. The raw data were converted into MGF format by Proteome Discoverer software (version 1.4) and then searched by Mascot search engine (version 2.3.01) against the UniProt-Homo proteome database. Search parameters were as following. Enzyme specificity was set as trypsin/P and up to two missed cleavages were allowed. Carbamidomethylation of cysteine was set as a fixed modification. The acetylation of protein N-term, oxidation of methionine and oxidation of proline were set as variable modifications. Mass tolerance of precursor ion was set as ±10 ppm, fragment mass tolerance was set as ±0.5 Da. The protein score was cut-off at 50.

### Mice experiment

*P4ha2* knockout mice were purchased from Cyagen Biosciences Inc. The gRNA to mouse *P4ha2* gene, and Cas9 mRNA were co-injected into fertilized mouse eggs to generate targeted knockout offspring. F0 founder animals were identified by PCR followed by sequence analysis, which were bred to wildtype mice to test germline transmission and F1 animal generation.

gRNA target sequences are shown in Supplementary Table 2.

Genotyping PCR Primers are shown in Supplementary Table 2.

C57BL/6 WT and *P4ha2*^*−/*^^−^ mice (6 weeks old, female) were randomly grouped and tail vein injected with 1 × 10^6^ Luciferased *Eµ-myc arf*^*−/*^^−^ B cells. Tumor volume was measured using an in vivo imaging system (IVIS). At the experimental endpoint, tumors were harvested and fixed with 4% PFA for paraffin-embedded section. Treatment with 40 mg/kg Ethyl 3,4-dihydroxybenzoate (EDHB, Sigma, E24859) or vehicle control (10% ethanol) started on day 2 after injection of Luciferased *Eµ-myc arf*^*−/*−^ B cells. All animal care and experimentation were ethically performed according to procedures approved by the Institutional Animal Care and Use Committee at Fudan University (20190221-001, 20220228-080).

### Human specimens

Symptomatic patient samples were collected as part of routine clinical examination. The study was conducted in accordance with the principles of the Declaration of Helsinki and was approved by institutional ethics committee under number 2022-K558, 2019-Y005. All patients provided written informed consent.

### RNA sequencing

RNA-seq was performed in isolated stromal fibroblasts from WT and *P4ha2*^*−/*^^−^ mice aged 8 weeks. Total RNA was extracted using the Trizol reagent according to the manufacturer’s protocol. RNA purity and quantification were evaluated using the NanoDrop 2000 spectrophotometer (Thermo Scientific, USA). RNA integrity was assessed using the Agilent 2100 Bioanalyzer (Agilent Technologies, Santa Clara, CA, USA). Then the libraries were constructed using TruSeq Stranded mRNA LT Sample Prep Kit (Illumina, San Diego, CA, USA) according to the manufacturer’s instructions. Briefly, 150 bp paired-end reads were processed and generated, the libraries were sequenced on an Illumina HiSeq platform. Differential gene expression analysis was done using the R package DEseq2(1.42.1). P value < 0.05 and log2 fold change > 0.2 or log2 fold change < -0.2 was set as the threshold for significantly differential expression. The Differential genes table is available in Supplementary Materials.

### Statistical analysis

The relative gene expression levels normalized by GAPDH were calculated by the formula 2 − ΔCt, where the ΔCt (critical threshold) = Ct of genes of interest −Ct of GAPDH. Statistical analysis was performed using the GraphPad Prism 8.0 software. Immunohistochemistry and immunoblot bands staining were measured by ImageJ. A two-tailed Student’s t-test was used to evaluate the group-level differences. Data were shown as the mean ± SEM. ****P < 0.0001, ***P < 0.001, **P < 0.01, *P < 0.05.

## Results

### P4HA2 forms an association with the Hedgehog key factor KIF7 and regulates the Hedgehog signaling

**Fig. 1:**
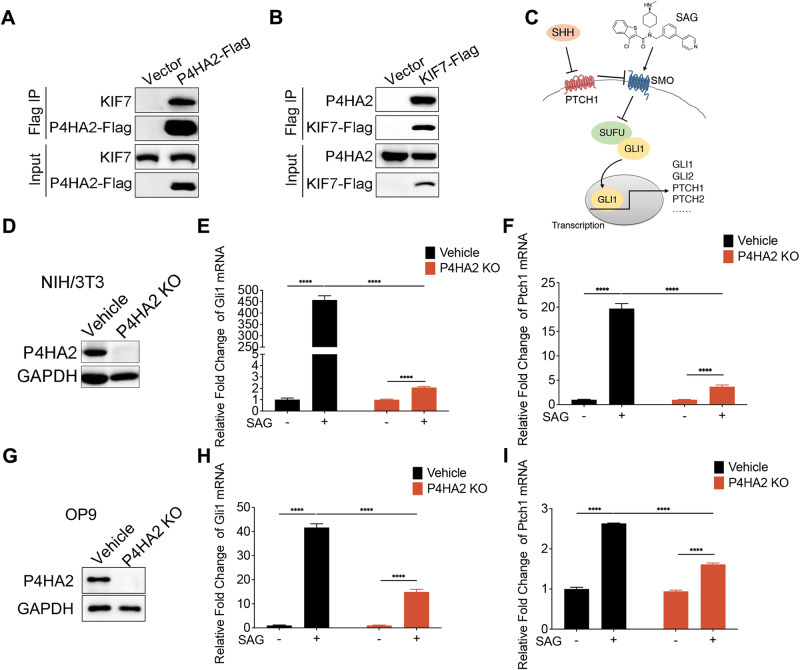
P4HA2 interacts with KIF7 and regulates the Hedgehog signaling. **A** P4HA2 interacts with KIF7. HEK293T cells were transfected with Flag-tagged P4HA2. Cell lysates were prepared and subjected to immunoprecipitation (IP) with M2 anti-Flag beads. The immunoprecipitates were analyzed by Western blotting (WB) with anti-Flag and KIF7 antibodies. **B** KIF7 interacts with P4HA2. HEK293T cells were transfected with Flag-tagged KIF7. Cell lysates were prepared and subjected to Flag IP. The immunoprecipitants were analyzed by WB with anti-Flag and P4HA2 antibodies. **C** Schematic representation of the SHH signaling pathway. SAG is a chlorobenzothiophene-containing Hh pathway agonist and binds to the SMO heptahelical bundle in a manner that antagonizes cyclopamine action. SAG activates SMO, leading to the release of GLI1 by SUFU, and the Hh signaling pathway is then activated. **D**–**I** Knockout of P4HA2 causes suppression of Hedgehog signal transduction. NIH/3T3 (**D**–**F**) and OP9 (**G**–**I**) P4HA2 knockout (KO) or vehicle cells were treated with (+) or without (−) 200 nM SAG. The Hh targeted genes, Gli1 (**E**, **H**) and Ptch1 (**F**, **I**), were analyzed by quantitative real-time polymerase chain reaction (qRT-PCR). Data are shown as the mean ± SEM (*n* = 3). *P < 0.05, **P < 0.01, ***P < 0.001, ****P < 0.0001. All experiments were repeated three times independently.

To delve into the function role of P4HA2, the Tandem Affinity Purification (TAP) method coupled with mass spectrometry (MS) was employed to identify enriched proteins in the TAP-P4HA2 immunoprecipitates (Supplementary Fig. 1A). Consistent with the known α2β2 heterotetramer structure of P4HA2, both α and β subunits were identified in the immunoprecipitates (Supplementary Table 1). KIF7, a kinesin-4 protein, is prominent among the newly identified P4HA2-binding proteins. To validate this interaction, we conducted an independent coimmunoprecipitation (Co-IP) assay, confirming the association between P4HA2 and KIF7 (Fig. 1A, B). To ascertain the specificity of this interaction, we explored the binding of KIF7 to P4HA1, a closely related isoform of P4HA2. The results indicated that KIF7 specifically interacts with P4HA2 (Supplementary Fig. 1B). Considering the pivotal role of KIF7 in the Hh pathway, we sought to assess the impact of P4HA2 on the Hh signaling in NIH/3T3 cells. We treated P4HA2 knockout cell lines with a Hh pathway activator, the SMO agonist SAG (Fig. 1C). Interestingly, in control cells, GLI1 and PTCH1 mRNA levels, the downstream targets of the Hh signaling pathway, significantly increased as expected upon SAG treatment. In contrast, the levels of GLI1 and PTCH1 mRNA in P4HA2 knockout cells were dramatically suppressed following SAG treatment (Fig. 1D–F). The inhibition of the Hh pathway activation by P4HA2 knockout were validated by different cell line clones (Supplementary Fig. 2A, B). Similarly, the P4HA2’s effect on Hh signal transduction was further validated in a variety of different cell lines (Fig. 1G–I and Supplementary Fig. 2C–E). Similar results were obtained when the pathway is activated upon the biological ligand Sonic Hedgehog (SHH) treatment (Supplementary Fig. 3).

Given the specific interaction between P4HA2 and KIF7, as opposed to its closely related isoform P4HA1, we sought to elucidate the role of P4HA1 in the Hh signaling. GLI1 luciferase activity was examined in cells with knockdown of either P4HA1 or P4HA2. Intriguingly, GLI1 luciferase activity was unchanged in P4HA1 knockdown cells, but was significantly inhibited in P4HA2 knockdown cells (Supplementary Fig. 4A–C). To further validate these results, we generated P4HA1 knockout cells (Supplementary Fig. S4D). In contrast to P4HA2 knockout cells, GLI1 transcription was modestly reduced in P4HA1 knockout cells (Supplementary Fig. 4E), consistent with the lack of detectable interaction between P4HA1 and KIF7 (Supplementary Fig. 1B). These findings underscore the specificity of the P4HA2-KIF7 interaction and highlight the distinctive roles of P4HA1 and P4HA2 in the Hh signaling. In summary, our results strongly support a specific interaction between P4HA2 and KIF7 and underscore the critical role of P4HA2 in the Hh signal transduction.

### P4HA2 migrates with KIF7 to regulate the Hedgehog pathway transduction

**Fig. 2:**
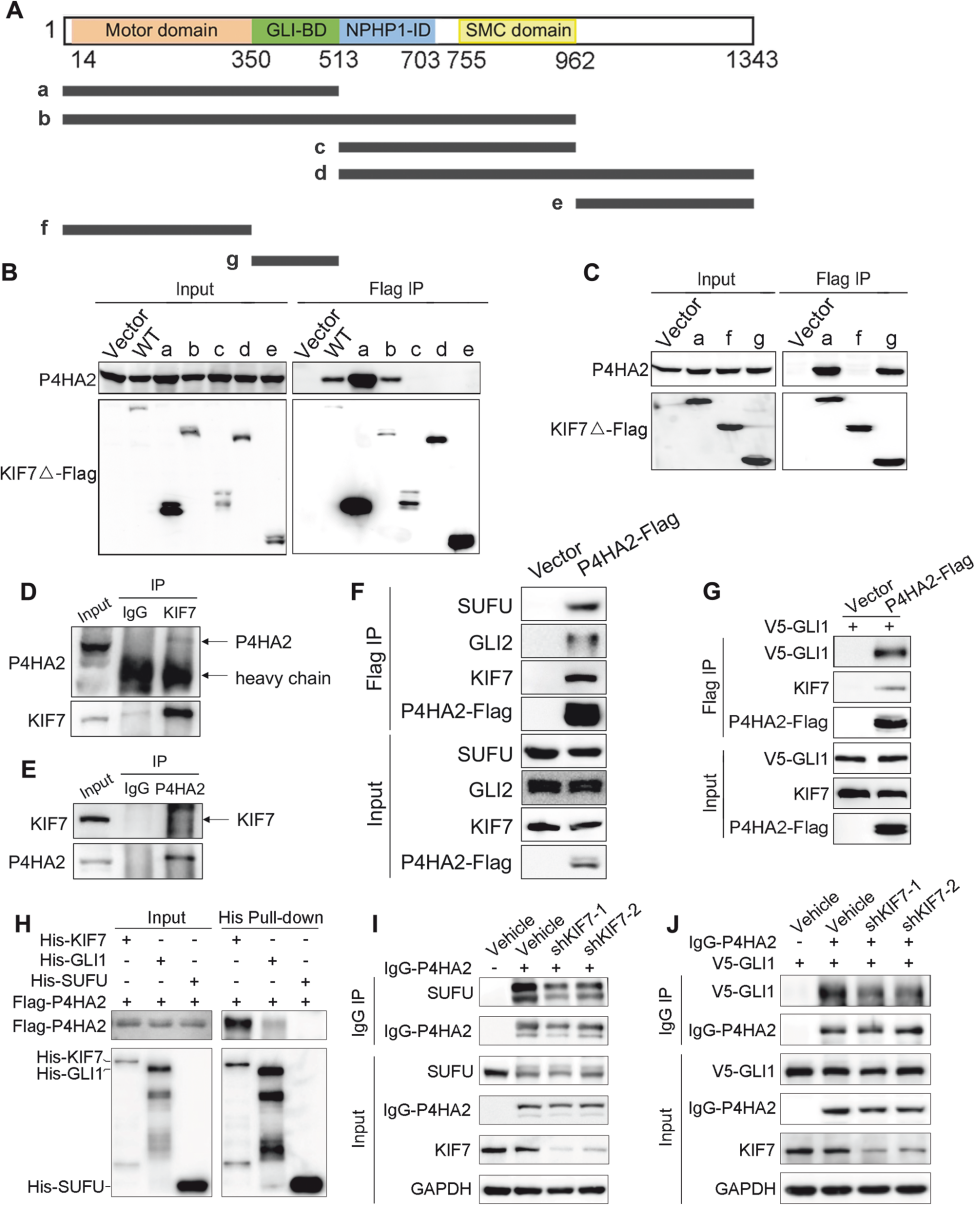
KIF7 mediates interaction between P4HA2 and the Hedgehog components. **A** Schematic of full-length KIF7 proteins. The KIF7 protein encodes a 1343 aa protein with a kinesin motor domain, GLI-binding domain (BD), Nephoronophthisis-1-interacting domain (NPHP1-ID) and a Structural Maintenance of Chromosomes (SMC) domain representing the ATPase activity. Various truncated mutation constructs of KIF7-Flag are shown schematically. **B** Mapping the interaction domain of KIF7 with P4HA2. HEK293T cells were transfected with constructs shown as (**A**). Cell lysates were prepared and subjected to immunoprecipitation (IP) with M2 anti-Flag beads. **C** Mapping the minimum interaction domain of KIF7 with P4HA2. HEK293T cells were transfected with constructs “a”, “f” and “g” mutants. Cell lysates were prepared and subjected to Flag IP. **D**, **E** Endogenous co-immunoprecipitated P4HA2 and KIF7 interact with each other separately in HEK293T cells. Endogenous KIF7 and P4HA2 were precipitated from a HEK293T cells extract and analyzed by immunoblotting. **F** P4HA2 interacts with SUFU and GLI2. HEK293T cells were transfected with Flag-tagged P4HA2. Cell lysates were subjected to Flag IP. The immunoprecipitants were analyzed by WB with anti-Flag, KIF7, SUFU and GLI2 antibodies. **G** P4HA2 interacts with GLI1. HEK293T cells were transfected with Flag-tagged P4HA2 and V5-tagged GLI1. Cell lysates were subjected to Flag IP. The immunoprecipitants were analyzed by WB with anti-Flag, KIF7 and V5 antibodies. **H** P4HA2 directly interacts with KIF7 in vitro. His-tagged KIF7, GLI1, SUFU, and Flag-tagged P4HA2 proteins were purified and incubated. After His pulldown, the complex was analyzed by WB. **I** KIF7 knockdown decreases the interaction between P4HA2 with SUFU. IgG-P4HA2 was overexpressed in shKIF7-1 and shKIF7-2 HEK293T cells. Cell lysates were subjected to IgG IP. Co-immunoprecipitated SUFU was detected by WB. **J** KIF7 knockdown decreases the interaction between P4HA2 and GLI1. IgG-P4HA2 and V5-GLI1 were co-overexpressed in KIF7 stable knockdown HEK293T cells (shKIF7-1 and shKIF7-2). Cell lysates were subjected to IgG IP. Co-immunoprecipitated V5-GLI1 was detected by WB.

To further characterize the interaction between P4HA2 and KIF7, we generated a series of Flag-tagged truncated mutants of KIF7 (Fig. 2A) and P4HA2 (Supplementary Fig. 5A). This analysis identified the “g” GLI-binding domain (GLI-BD) of KIF7 and “h”, including protein-substrate binding (PSB) domain and part of CAT domain of P4HA2, are crucial for the interaction between KIF7 and P4HA2 (Fig. 2B, C; Supplementary Fig. 5B). To further determine the physiological interaction between P4HA2 and KIF7, we carried out endogenous co-IP assays. First, we enriched KIF7 protein with a specific antibody and detected P4HA2 in the KIF7 antibody immunoprecipitates (Fig. 2D). Moreover, the reverse co-IP assay also yielded positive results (Fig. 2E). We also co-transfected the corresponding interaction domains of P4HA2 and KIF7, and successfully purified the complex in vitro, which further confirmed that they were able to form a tight complex (Supplementary Fig. 5C). Given that the GLI-BD of KIF7 mediates its interaction with GLI and is involved in organizing cilia tip compartments for the regulation of the SUFU-GLI complex, it warranted to investigate whether P4HA2 associates with SUFU-GLI complex via KIF7. To ascertain whether GLIs or SUFU associates with P4HA2, we carried out co-IP assays. SUFU, GLI2, V5-GLI1 (as endogenous GLI1 protein expression is barely detected in the absence of the Hh signaling) and KIF7 were detected in the P4HA2 immunoprecipitates (Fig. 2G, H). Subsequently, a pulldown experiment using purified protein was performed to further characterize the interactions. Notably, a strong direct interaction was observed between P4HA2 and KIF7, while no significant or direct interaction was detected between P4HA2 and GLI1 or SUFU (Fig. 2F). These results imply that the formation of complexes involving SUFU, GLI1/2, KIF7 and P4HA2, while P4HA2 directly associates with KIF7. To investigate whether these interactions are perturbed in the KIF7-depleted condition, we examined the immunoprecipitates in cells with KIF7 knockdown. The amount of V5-GLI1 or SUFU protein in the IgG-P4HA2 immunoprecipitates was significantly decreased in cells with reduced KIF7 expression compared to the control (Fig. 2I, J), underscoring the crucial role of KIF7 in mediating the interaction between P4HA2 and GLI1, as well as P4HA2 and SUFU.

**Fig. 3:**
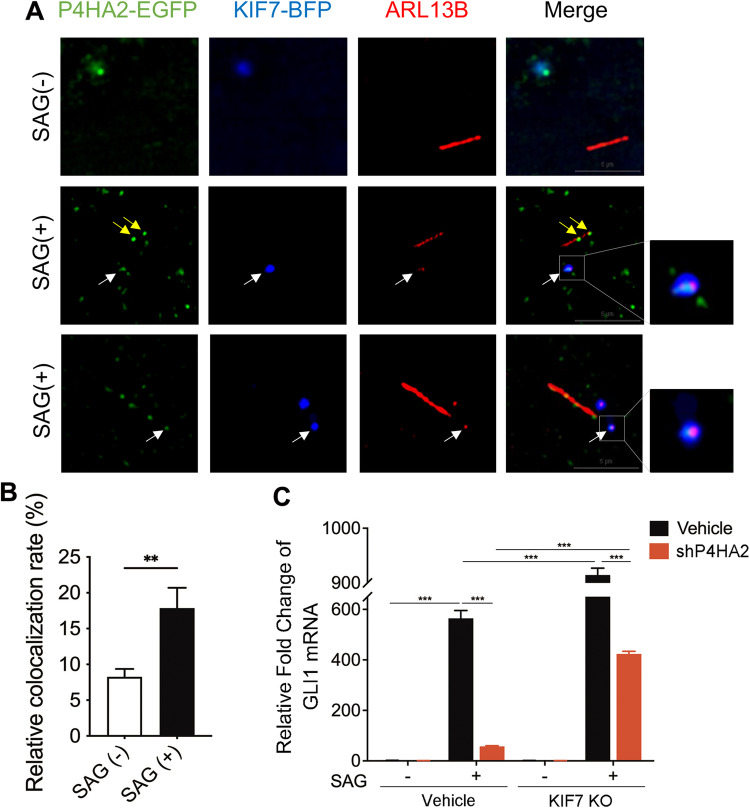
P4HA2 migrates with KIF7 to regulate the Hedgehog pathway transduction. **A** The trafficking of the P4HA2-KIF7 complex from the cytoplasm to the cilium responds to Hedgehog signaling. NIH/3T3 cells were co-infected with KIF7-BFP and P4HA2-EGFP lentivirus. White arrow indicates the co-localization of cilia, KIF7-BFP and P4HA2-EGFP. Yellow arrow indicates the co-localization of cilia and P4HA2-EGFP, which may bind with endogenous KIF7. Cells were treated with 200 nM SAG (+) or not (−). Cells were fixed and stained with antibodies for ARL13B to mark primary cilia. Scale bars: 5 μm. **B** Statistical analysis of the relative colocalization rate in (**A**) indicated cells. Data are shown as the mean ± SEM; SAG (−), *n* = 11; SAG (+), *n* = 13. **P < 0.01. **C** The regulation of the Hedgehog signaling by P4ha2 is dependent on KIF7. KIF7 knockout (KO) and the control cells were knocked down with P4HA2 (shP4HA2). Cells were treated with (+) or without (−) 200 nM SAG. qRT-PCR analysis of GLI1 transcripts was performed using RNA isolated from the indicated cell lines. Data are shown as the mean ± SEM (*n* = 3). ***P < 0.001. All experiments were repeated three times independently.

KIF7 forms dynamic complexes with core components of the Hh pathway, and its trafficking from the base to the tip of the cilium is promoted upon the Hh signaling activation [4]. To investigate the mechanism underlying P4HA2’s new function in the Hh pathway, we examined the localization of P4HA2 at different stages of the Hh pathway activation. We tagged P4HA2 and KIF7 with EGFP and BFP fluorescence proteins, respectively, and then transfected cells. We found that P4HA2-EGFP co-localized with KIF7-BFP in cytoplasm. Upon SAG treatment, P4HA2-EGFP, KIF7-BFP and ARL13B, a marker of cilia, were co-localized together. Furthermore, the P4HA2-EGFP-KIF7-BFP complex appeared to be on the tips of the cilia (Fig. 3A, B). In time-lapse imaging, SAG treatment induced the migration of the P4HA2-KIF7 complex from cytoplasm to cilium, moving along with the microtubules of cilium (Supplementary Video 1). These results suggested that the ciliary fraction of P4HA2-KIF7 complex significantly increased after SAG treatment. Notably, P4HA2-EGFP relocated onto cilia after SAG treatment, which may be due to the interaction between P4HA2-EGFP and endogenous KIF7. To this end, we constructed KIF7 knockout cells (Supplementary Fig. 6A). Cilia in KIF7 null cells exhibited a long and irregular morphology, consistent with previous reports [4]. Using a P4HA2 fused with mIFP to track its localization (KIF7 knockout cells were green), we observed that P4HA2-mIFP was not observed in the primary cilia, either presence or absence of SAG treatment in the KIF7 knockout cells (Supplementary Fig. 6B), suggesting that the ciliary localization of P4HA2 is dependent on KIF7.

Considering the reliance of P4HA2’s interaction with GLI1/SUFU and ciliary localization on KIF7, we investigate whether the effects of P4HA2 on the Hh signaling depends on KIF7. We conducted P4HA2 knockdown in both KIF7 knockout and control cells (Supplementary Fig. 6C). Our results revealed that P4HA2 knockdown blocked the increased GLI1 mRNA levels upon SAG treatment. Surprisingly, the inhibitory effect of P4HA2 knockdown on GLI1 transcription was partially rescued in KIF7 knockout cells compared with vehicle control cells. This suggests that the influence of P4HA2 on the Hh signaling is modulated by KIF7 (Fig. 3C). In summary, KIF7 serves as a scaffold protein, facilitating the ciliary localization of P4HA2 and its association with other components, thereby regulating the impact of P4HA2 on the Hh signaling pathway.

### Hydroxylation of SUFU by P4HA2 mediates the Hedgehog pathway transduction

**Fig. 4:**
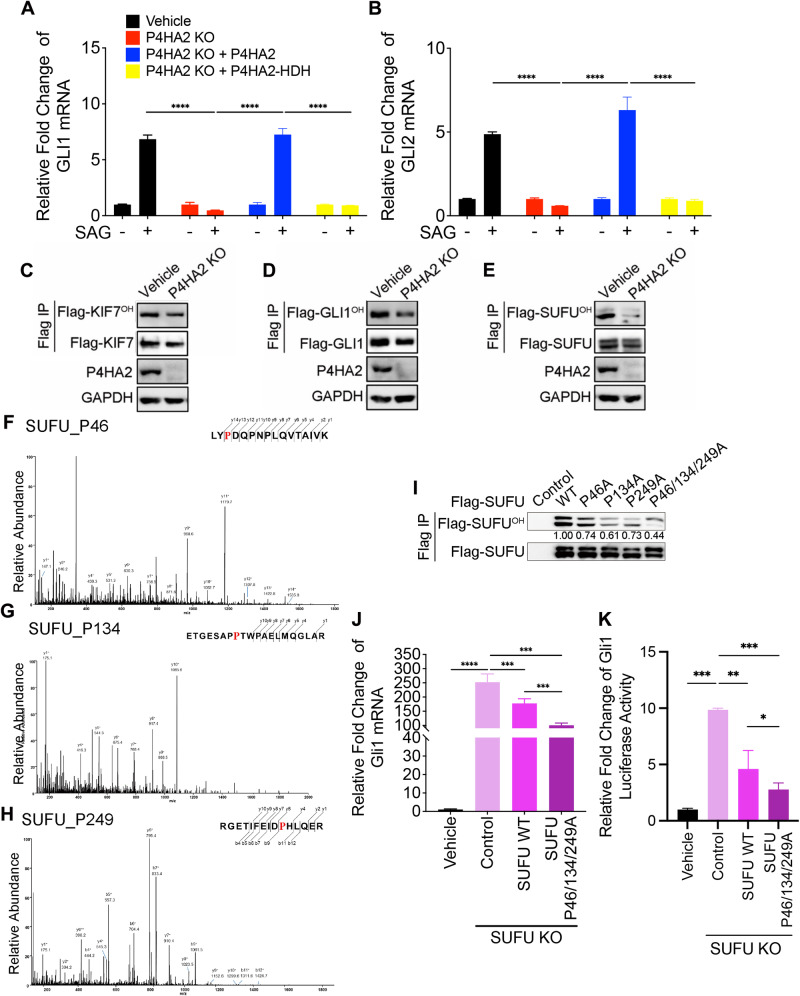
P4HA2 hydroxylates SUFU to regulate the Hedgehog signaling. **A**, **B** The regulation of Hh signaling by P4HA2 is dependent on P4HA2 hydroxylase activity. HEK293T P4HA2 KO cells were expressed with wild-type P4HA2 (P4HA2) or hydroxylase dominant negative mutant (P4HA2-HDH). Cells were treated with (+) or without (−) 200 nM SAG. qRT-PCR analysis of GLI1 (**A**) and GLI2 (**B**) transcripts was performed using RNA isolated from the indicated cell lines. Data are shown as the mean ± SEM (*n* = 3). *P < 0.05, ***P < 0.001. All experiments were repeated three times independently. **C**–**E** The hydroxylation levels of GLI1 and SUFU decrease in P4HA2 knockout cells. P4HA2 KO and the vehicle cells were transfected with Flag-tagged KIF7, GLI1, and SUFU. Cell lysates were subjected to Flag IP and then analyzed by WB. Hydroxylated proteins were recognized by the Pan-hydroxylation antibody (OH). The relative quantification of Flag-KIF7 (**C**), GLI1 (**D**), and SUFU (**E**) hydroxylation level was analyzed and normalized. The values shown are the means of three independent experiments. **F**–**H** LC-MS/MS spectra of tryptic peptides. 3 tryptic peptides Pro 46 (**F**), Pro 134 (**G**), and Pro 249 (**H**) from SUFU. The b ion and y ion are fragment ions of tryptic peptide in tandem mass spectrometry. The x and y axes represent m/z and relative ion intensity, respectively. **I** Validation of the proline 46, proline 134, and proline 249 hydroxylation sites of SUFU. P46A, P134A, P249A, and P46/134/249A proline to alanine mutants of SUFU were constructed and transfected into HEK293T cells. Cell lysates were subjected to Flag IP. The relative quantification of SUFU WT and mutant hydroxylation levels was analyzed and normalized. The values shown are the means of three independent experiments. **J** P4HA2 regulates the protein stability of SUFU in response to the Hh pathway activation. HEK293T P4HA2 KO cells were expressed with wild-type P4HA2 or a P4HA2 dominant negative mutant in hydroxylase activity (HDH). Cell lysates were analyzed by WB with anti-P4HA2 and GAPDH antibodies. **K** Hydroxylated SUFU regulate the Hh signaling. SUFU knockout (KO) NIH/3T3 and control cell lines were constructed and then infected with SUFU WT and mutant (SUFU P46/134/627 A) lentivirus. qRT-PCR analysis of GLI1 transcripts was performed using RNA isolated from the above cell lines. **L** SUFU KO NIH/3T3 and control cell lines were constructed and then infected with SUFU WT and mutant (SUFU P46/134/627 A) lentivirus. Utilized a dual luciferase reporter gene system, GLI1-Firefly and Renilla plasmids were overexpressed in above cell lines. Relative fold change of GLI1 luciferase activity was measured and normalized. Data are shown as the mean ± SEM (*n* = 3). ***P < 0.001, ****P < 0.0001. All experiments were repeated three times independently.

To determine whether the function of P4HA2 in the Hh pathway relies on its hydroxylase activity, we conducted rescue experiments. We examined the ability of wild-type P4HA2 and its hydroxylase-deficient mutant (P4HA2-HDH) to restore the Hh signaling in P4HA2 knockout cells. Upon SAG stimulation, GLI1 and GLI2 mRNA were upregulated in the P4HA2 wild-type rescue groups (P4HA2 KO + P4HA2) but not in the P4HA2 hydroxylase-deficient mutant rescue group (P4HA2 KO + P4HA2-HDH) (Supplementary Fig. 7A; Fig. 4A, B). This suggests that the hydroxylase activity of P4HA2 is essential for its function in the Hh pathway. To identify potential substrates of P4HA2 involved in the Hh signaling, we examined the hydroxylation levels of three P4HA2-interacting proteins-KIF7, GLI1, and SUFU-in both P4HA2 wild-type and knockout cells. The hydroxylation level of SUFU was markedly downregulated in P4HA2 knockout cells comparing with the KIF7 or GLI1 group (Fig. 4C–E). These results suggest that SUFU is the putative substrate for P4HA2. We then conducted MS analysis to identify the putative hydroxylated sites on SUFU. The 46^th^, 134^th^, 249^th^ and 482^th^ proline residues of SUFU showing decreased hydroxylation levels in P4HA2 knockout cells compared with the control were considered as the potential positive signals. These signals were further validated by examining the hydroxylation levels using site-directed mutagenesis (proline to alanine, P-A) (Supplementary Fig. 7B). Hydroxylated modifications were successfully identified and confirmed for Pro-46, 134 and 249 of SUFU (Fig. 4F–H). We generated a triple-site mutant, SUFU P46/134/249 A, which exhibited lower hydroxylation levels compared with single-site mutants or the wild-type control (Fig. 4I).

To investigate the impact of SUFU hydroxylation by P4HA2 on the Hh signaling, we circumvented the challenge posed by the high levels of endogenous SUFU by generating a SUFU knockout cell line for the overexpression of SUFU wild-type (WT) and mutant constructs (Supplementary Fig. 7C). In SUFU knockout cells, GLI1 mRNA levels were greatly elevated, and this phenotype was partially rescued by SUFU WT overexpression. Notably, overexpression of the SUFU P46/134/249 A mutant led to a more robust suppression of GLI1 transcript expression (Fig. 4J). Meanwhile, we utilized a dual luciferase reporter gene system, which is more responsive to true function, to overexpress GLI1-Firefly and Renilla plasmids in four types of cells above to further verify the hypothesis (Fig. 4K). These results highlight that P4HA2 modulates the Hh signaling pathway through the hydroxylation of SUFU. In summary, SUFU has been identified as the substrate of P4HA2, and the hydroxylation of SUFU by P4HA2 plays a crucial role in mediating the modulation of P4HA2 on the Hh signaling transduction.

### P4HA2 promotes B-cell lymphoma through a paracrine signaling transduction manner

To further detect the modulation of P4HA2 on the Hh pathway transduction in vivo, we employed a CRISPR/Cas9 strategy to generate *P4ha2* knockout mice, which covered all *P4ha2* exons (Supplementary Fig. 8A). Due to the significant regulation of the Hh pathway on development, we also examined whether the development of *P4ha2*^*−/*^^−^ mice would be affected. We found that the birth rate of *P4ha2*^*−/*^^−^ mice was lower than that of wild-type and heterozygous mice (Supplementary Fig. 8B), suggesting potential abnormalities during embryonic development. Further examination revealed defects in few *P4ha2*^*−/*^^−^ mice during the embryonic development stage, as confirmed by genotype identification (Supplementary Fig. 8C, D). We examined Gli1 expression in embryos by immunohistochemical (IHC) staining. As shown in Supplementary Fig. 8E, F, Gli1 expression in the *P4ha2*^*−/*^^−^ embryo was significantly lower than that in the wildtype embryo. These results indicate that P4HA2 is involved in the regulation of embryonic development processes mediated by the Hh pathway.

**Fig. 5:**
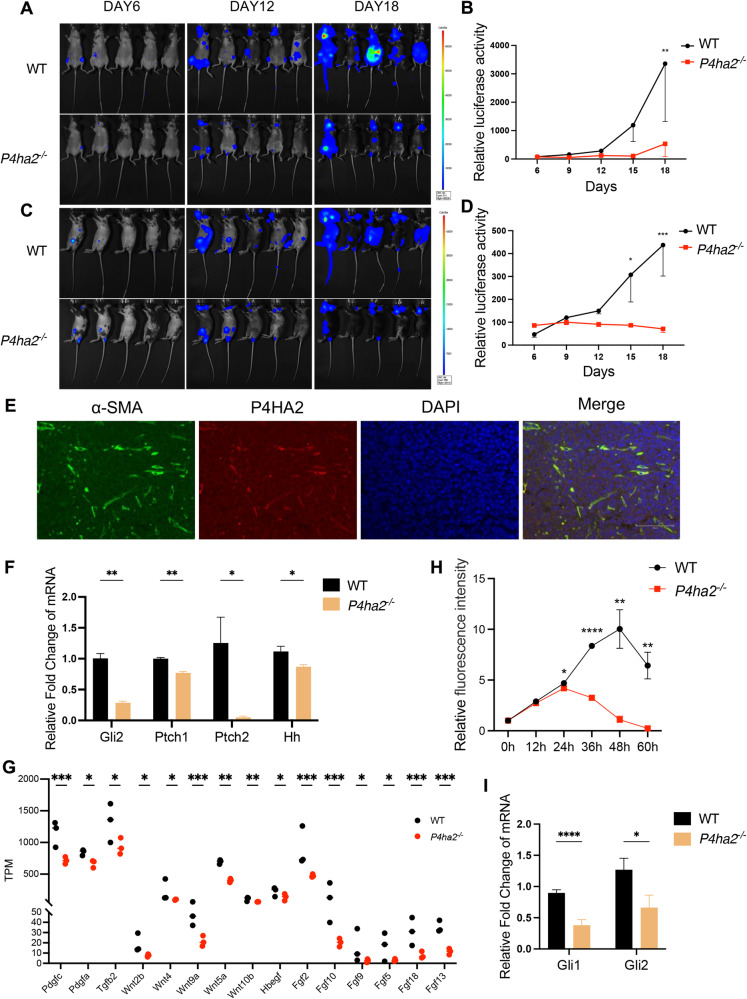
Promotion of B-cell lymphoma tumorigenesis by P4HA2 in stromal fibroblasts. **A**–**D** Injection of luciferased *Eµ-myc arf*^*−/*^^−^ B cells into C57BL/6 WT and *P4ha2*^*−/*^^−^ mice. Bioluminescence imaging (**A**, **C**) of mice for 18 days after injection. Luminescence intensity curves (**B,** **D**) for mice injected with luciferased *Eµ-myc arf*^*−/*^^−^ B cells. **E** P4HA2 is located in stromal fibroblasts. Immunofluorescence staining of α-SMA, P4HA2 and DAPI in C57BL/6 WT mice xenograft. Scale bars: 100 μm. **F** Primary stromal fibroblasts were isolated from bone marrow of C57BL/6 WT and *P4ha2*^*−/*^^−^ mice. qRT-PCR analysis of Gli2, Ptch1, Ptch2 and HH ligands transcripts was performed using RNA isolated from the primary stromal fibroblasts. **G** Differential genes of Hh downstream secreted factors obtained from RNA-seq analysis of primary bone marrow stromal fibroblasts. Data are shown as the mean ± SEM (*n* = 3). *P < 0.1, **P < 0.01, ***P < 0.001. **H** The growth curve of *Eµ-myc arf*^*−/*^^−^ B cells co-cultured with primary bone marrow stromal fibroblasts supernatant. WT and *P4ha2*^*−/*^^−^ primary bone marrow fibroblasts were isolated and cultured. After cell counting, both primary cells were cultured at the same density, and the cultured supernatant was collected for co-culture with *Eµ-myc arf*^*−/*^^−^ B cells. The cell viability was detected every 12 hr. **I** qRT-PCR analysis of Gli1 and Gli2 transcripts was performed using the tissue of C57BL/6 WT and *P4ha2*^*−/*^^−^ mice xenograft. Data are shown as the mean ± SEM (*n* = 3). ***P < 0.001, ****P < 0.0001. All experiments were repeated three times independently.

Aberrant activation of the Hh signaling pathway is observed in B-cell lymphoma tumorigenesis through a manner of paracrine signaling. The malignance proliferation of tumor cells relies on Hh signals emanating from stromal cells. Notably, P4HA2 was highly expressed in tumor stromal fibroblasts in DLBCL samples (Supplementary Fig. 9A-B), which promoted us to investigate the role of the P4HA2-Hh axis in stromal cells promoting B-cell lymphoma tumorigenesis. Luciferase-expressing *Eµ-myc arf*^*−/*^^−^ B lymphoma cells, exhibiting very low P4HA2 expression (Supplementary Fig. 10A), were injected via the tail vein into both *P4ha2* knockout and wildtype mice. Thus, wildtype mice harbored P4ha2 mainly in stromal cells. Notably, 18 days post-injection, wildtype mice exhibited high luminescence in the lymph nodes and spleens, while *P4ha2* knockout mice showed minimal luminescence (Fig. 5A-D). The overall survival of *P4ha2* knockout mice increased to 29 days compared to 26 days in wildtype mice (Supplementary Fig. 10B). Immunofluorescence analysis of tumors isolated from wildtype mice confirmed co-localization of P4HA2 and α-SMA, a fibroblast marker (Fig. 5E), which is similar with DLBCL tumor samples (Supplementary Fig. 9A). These results demonstrate that high expression of P4HA2 in stromal fibroblast dominantly prompts B-cell lymphoma tumorigenesis.

Considering the reported substrates of P4HA2, it’s noteworthy that Carabin is not expressed in fibroblasts, whereas collagen is. We assessed collagen levels through Sirius Red staining, revealing minimal differences between wildtype and *P4ha2* knockout mice xenograft (Supplementary Fig. 10C). This observation suggests that collagen and Carabin may not be the primary factors influencing stromal P4HA2’s impact on B-cell lymphoma progression. The stromal Hh signaling is crucial for survival signal for B-cell malignancies [25], we explored the role of P4HA2 expressed in stromal fibroblast in the Hh pathway. We detected the Hh downstream signal mRNA levels in spleen and bone marrow stromal fibroblasts between wildtype and *P4ha2* knockout mice. The results revealed a significant reduction in the transcription of Gli and Ptch. In addition, the Hh ligand mRNA levels were also decreased upon potential positive feedback regulation [42] (Fig. 5F; Supplementary Fig. 11A). We further validated the inhibition in protein levels. Both full-length in cell lysis and cleaved SHH in supernatant were downregulated in P4HA2 knockout stromal cells (Supplementary Fig. 11B, C). This suggests that P4HA2 promotes the Hh production in stromal fibroblast. For the Hh downstream target genes, we utilized RNA seq analysis to characterize stromal fibroblasts from P4HA2 knockout compared to wildtype mice. The results revealed significant decreases in reported Hh pathway downstream target genes, such as Fgfs and Wnts (Fig. 5G), which are growth factors critical in the stromal-to-tumor signaling [43–48]. To validate the roles of stromal fibroblasts paracrine signaling in B lymphoma cells, we constructed a co-culture system and investigated if the activation of Hh signaling induced by stromal fibroblast based on the expression of P4HA2. Co-culturing *Eµ-myc arf*^*−/*^^−^ B lymphoma cells with *P4ha2* knockout stromal fibroblast supernatant resulted in a deceleration of cell proliferation compared to the control group (Fig. 5H). Noting that SHH declined in P4HA2 knockout stromal cells, we added exogenous SHH protein in the co-culture system and found that the proliferation inhibition was reversed (Supplementary Fig. 11D). Meanwhile, we examined the Hh pathway in tumor xenograft tissues and observed a significant decrease in the levels of the downstream targets of Hh pathway, Gli1 and Gli2 (Fig. 5I). These findings suggest that P4HA2 plays a role in the production of the Hh downstream growth factors and HH ligands by stromal fibroblast, affecting the proliferation of B lymphoma cells.

Furthermore, we examined human DLBCL pathological serial section, and confirmed that P4HA2 and KIF7 are both expressed in stromal fibroblasts (Supplementary Fig. 12A). Furthermore, stromal P4HA2 expression was significantly correlated with the expression of GLI2 in most DLBCL specimens (Supplementary Fig. 12B). Afterwards, we downloaded RNA-Seq and clinical data of TCGA-DLBC project from The Cancer Genome Atlas (TCGA) database. Our analysis revealed a significant correlation between the expression levels of Hh downstream target genes, including GLI2, TWIST2, SNAI1, JAG2 and others [23], with P4HA2 expression in higher grade DLBCL samples (Supplementary Fig. 12C). Additionally, treatment with C-P4H inhibitor, Ethyl 3,4-dihydroxybenzoate (EDHB), phenocopied P4HA2 knockout effects, as shown in Supplementary Fig. 13A, B, indicating that EDHB targets P4HA2 in stromal fibroblast to delay B-cell lymphoma progression. These data collectively indicate that P4HA2 expressed in stromal fibroblast can promote B-cell lymphoma progression via regulating the Hh signaling in a paracrine signal transduction manner.

## Discussion

**Fig. 6:**
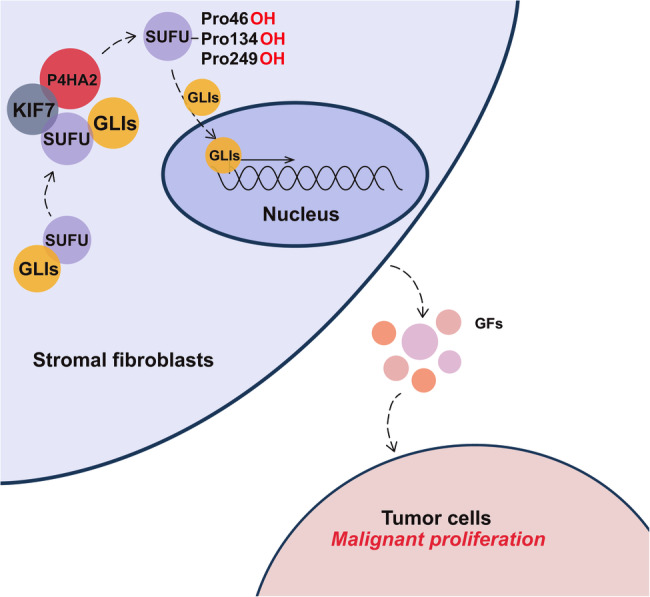
Schematic of P4HA2 promoting B-cell lymphoma through regulating the Hedgehog pathway in a paracrine transduction manner. In stromal cells, P4HA2 hydroxylates SUFU to regulate the Hh pathway. This leads to the activation of Hh signaling and the secretion of downstream growth factors, resulting in the malignant proliferation of tumor cells.

The findings presented in this study unveil a previously unrecognized role of P4HA2 in the regulation of the Hh signaling pathway and its consequential impact on B-cell lymphoma tumorigenesis via a paracrine mechanism. Through a series of comprehensive experiments, we have elucidated that P4HA2 is transported to the tips of primary cilia by KIF7, where it hydroxylates SUFU. This hydroxylation downregulates the function of SUFU, leading to the release of GLI and the subsequent transcriptional activation of downstream reporter genes. This paracrine signaling cascade, targeting B lymphoma cells, emerges as a key promoter of tumorigenesis (Fig. 6).

The P4HA2-KIF7 interaction uncovered in our study provides novel insights into the dynamic regulation of the Hh signaling cascade. Despite the previous CRISPR-based screenings of the Hh pathway regulators [5, 49], where P4HA2 was not uncovered, three independent proteome interactome studies have detected the interaction between P4HA2 and KIF7 [50–52]. These findings support our conclusion that P4HA2 is a novel component of the Hh pathway via associating with KIF7. The specificity of the P4HA2-KIF7 interaction is emphasized by the absence of similar interactions with the closely related isoform P4HA1. Our results underscore the unique contribution of P4HA2 in regulating Hh pathway activity when compared to P4HA1.

The con-trafficking of P4HA2 and KIF7 from the cytoplasm to cilia in response to the Hh signals, leading to the hydroxylation of SUFU, enriches our understanding of the Hh pathway. Proline hydroxylation by P4HA2 emerges as a new and distinct post-translational modification critical for pathway activation. Previous studies have implicated PKA, CK1, and GSK3 in the regulation of SUFU stability and localization through their kinase activities [53]. The identification of SUFU as a substrate for P4HA2-mediated hydroxylation contributes significantly to our understanding of the post-translational modifications governing the Hh signaling. Our results indicate that P4HA2-mediated hydroxylation SUFU, influencing the dynamics of the SUFU-GLI complex. The hydroxylation sites on SUFU, specifically Pro-46, 134, and 249, were identified through MS analysis, and the functional importance of these sites was confirmed through site-directed mutagenesis. Additionally, KIF7, acting as a key scaffold protein, is involved in the post-translational modification of the Hh pathway component, facilitating the hydroxylation of key proline residues in SUFU.

The mechanism of P4HA2-mediated hydroxylation leading to substrate degradation through the ubiquitin-proteasome system has been established in previous findings and is consistent with examples like Carabin or HIF1α regulation by hydroxylation under normoxia [26, 38]. However, other substrates—such as collagen and the virus proteins ZIKV and DENV—are not degraded after hydroxylation modification. In this case, C-P4Hs act as co-translational factors to modify the substrates, thereby enhancing their stability [54, 55]. The different roles may depend on the different subcellular locations of P4HA2 and its substrates. C-P4Hs in the endoplasmic reticulum appear to play a positive role in maintaining substrate stability. The observed changes in SUFU function correlated with P4HA2 expression, but further investigation is needed to investigate whether substrate degradation upon hydroxylation by P4HA2 occurs through the ubiquitin-proteasome system.

The Hh pathway has served as a promising therapeutic target for various cancers, with SHH, SMO, and GLI validated as targetable factors. However, severe adverse effects and drug resistance limit the clinical utility. P4HA2, as an enzyme that precisely regulates the Hh pathway transduction, presents itself as a suitable intervention target. Our murine model with *P4ha2* knockout demonstrates the physiological relevance of P4HA2 in B-cell lymphoma progression, highlighting its crucial role in the stromal microenvironment. The observed downregulation of Gli and growth factors in the *P4ha2* knockout stromal cells, suggests that P4HA2 intricately modulates Hh paracrine signaling. Integrating findings from a prior study wherein P4HA2 was identified as promoting B-cell lymphoma in tumor cells, and the current study revealing its high expression in the stromal fibroblasts of B-cell lymphoma, emphasizes P4HA2 as a promising target for therapeutic intervention.

In conclusion, our study uncovers a novel regulatory axis involving P4HA2, KIF7, and SUFU in the Hh signaling pathway, shedding light on the intricate mechanisms governing B-cell lymphoma tumorigenesis. The identification of P4HA2 as a key player in stromal fibroblasts emphasizes its potential as a therapeutic target for disrupting the tumor microenvironment and influencing Hh signaling in B-cell lymphoma. Further investigations into the specific P4HA2 inhibitors may offer an attractive new therapeutic strategy for B-cell lymphoma.

### Supplementary information

Supplemental Table

Supplemental Video

Supplemental Data

## Data Availability

The data that support the findings of this study are available from the corresponding author upon reasonable request. Differential gene expression analysis of RNA-Seq was enclosed in Supplementary Material.
