# Supplementary material for: P4HA2 hydroxylates SUFU to regulate the paracrine Hedgehog signaling and promote B-cell lymphoma progression
Source: Leukemia. 2024 Jun 22;38(8):1751–63. doi: 10.1038/s41375-024-02313-8 (PMC11286522; doi:10.1038/s41375-024-02313-8)
Supplement: Supplementary file 3 — Supplemental Data [file 41375_2024_2313_MOESM3_ESM.docx]

**Supplemental Data**

**Supplemental Figures**


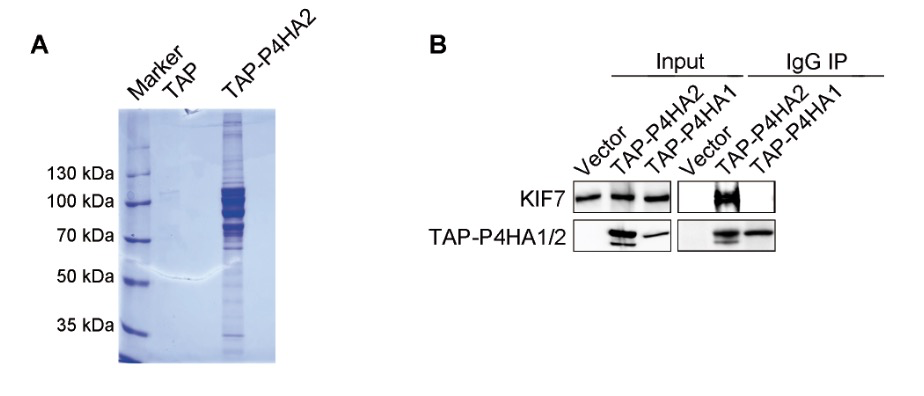


**Supplemental** **Figure 1. KIF7 is identified as a P4HA2-associated protein.** (A) Pulldown of P4HA2 by tandem affinity purification. Bands from Coomassie staining were sent for protein identification by mass spec (MS). (B) KIF7 combines with P4HA2, not P4HA1. TAP-P4HA1 and TAP-P4HA2 were expressed and cell lysates were prepared and subjected to IP with anti-IgG beads.


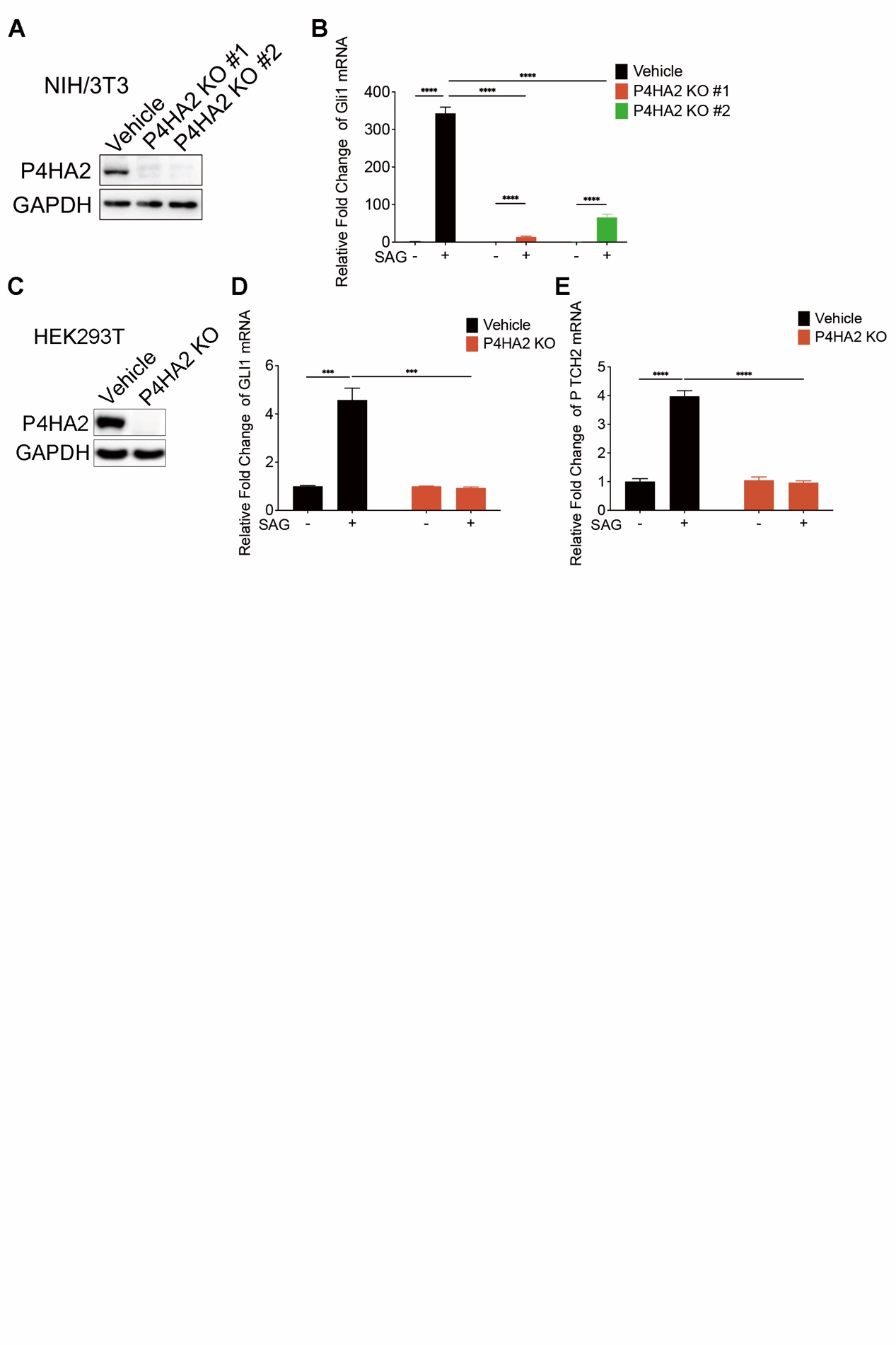


**Supplemental** **Figure 2. Knockout of P4HA2 suppresses the Hh signaling in human cells.** (A) Multiple clones of P4HA2 knockout NIH/3T3 cells were constructed. (B) NIH/3T3 knockout (KO) or vehicle cells were treated with (+) or without (-) 200 nM SAG. The Hh targeted genes, Gli1 was analyzed by quantitative real-time polymerase chain reaction (qRT-PCR). (C) P4HA2 knockout HEK293T cell line was constructed. (D-E) HEK293T knockout (KO) or vehicle cells were treated with (+) or without (-) 200 nM SAG. The Hh targeted genes, GLI1 (D) and PTCH2 (E), were analyzed by qRT-PCR. Data are shown as the mean ± SEM (n=3). *P < 0.05, **P < 0.01, ***P < 0.001, ****P < 0.0001. All experiments were repeated three times independently.


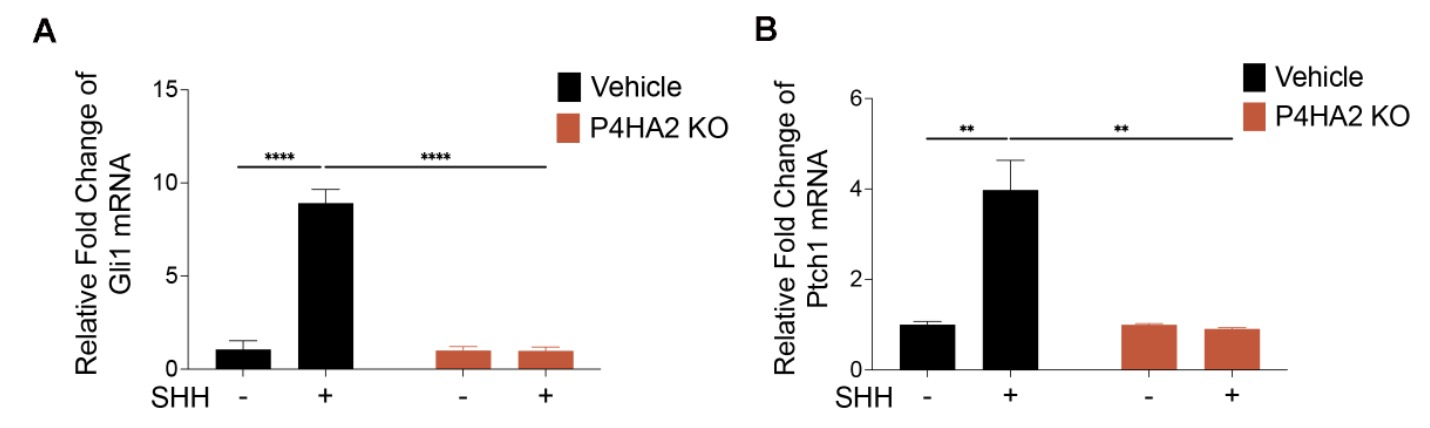


**Supplemental** **Figure 3. Knockout of P4HA2 inhibits the Hh pathway transduction in response to the Hh ligand.** (A-B)**,** NIH/3T3 P4HA2 knockout (KO) or vehicle cells were treated with (+) or without (-) 200ng/mL SHH. The Hh targeted genes, Gli1 (A) and Ptch1 (B), were analyzed by qRT-PCR. Data are shown as the mean ± SEM (n=3). *P < 0.05, **P < 0.01, ***P < 0.001, ****P < 0.0001. All experiments were repeated three times independently.

**
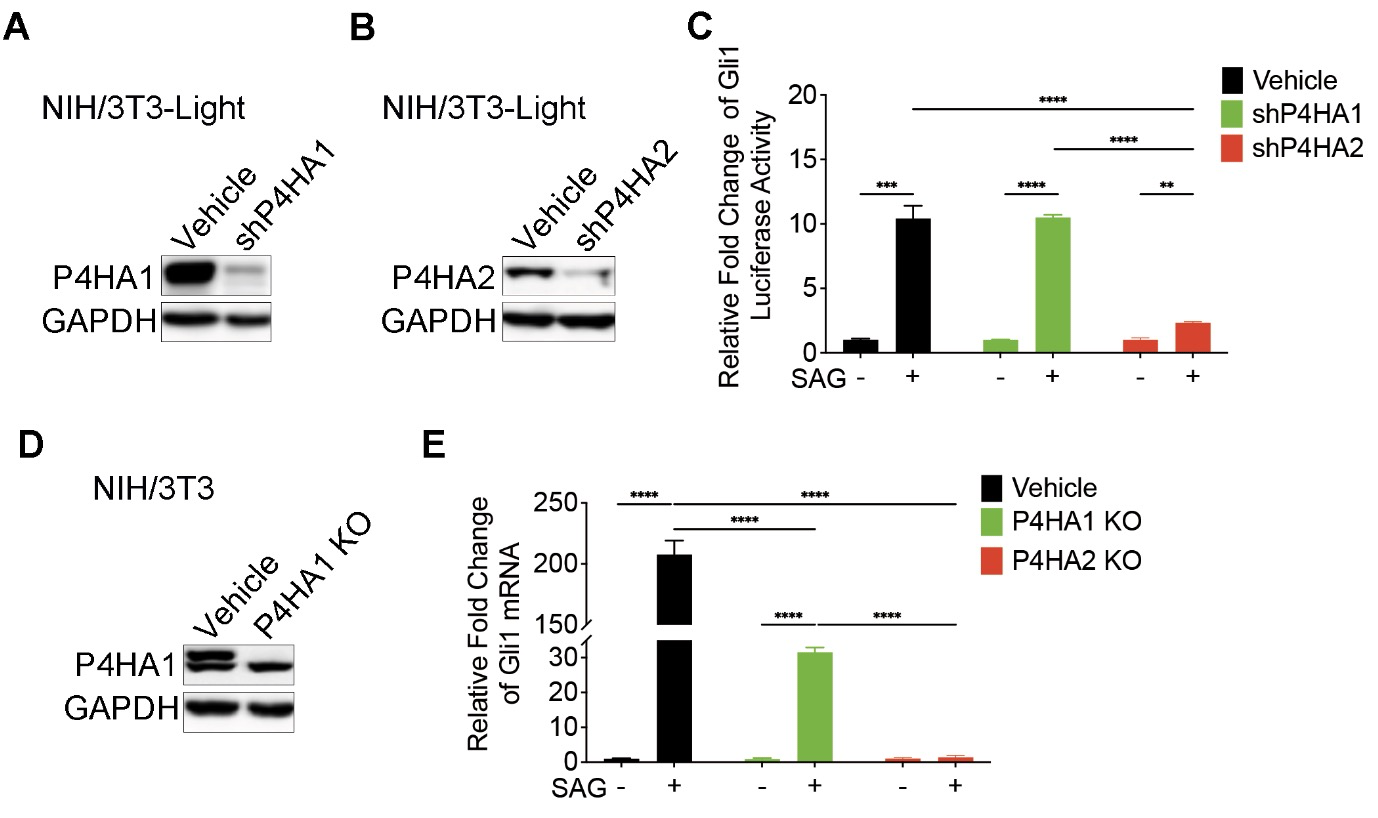
**

**Supplemental** **Figure 4. P4HA2, not P4HA1, is a specific regulator of the Hh signaling.** (A-B) P4HA1 (A) and P4HA2 (B) knockdown NIH/3T3 Gli1-luciferased cell lines were constructed. (C) NIH/3T3 Knockdown or vehicle cells were treated with (+) or without (-) 200 nM SAG. Relative fold change of GLI1 luciferase activity was measured and normalized. (D) P4HA1 knockout NIH/3T3 cell line was constructed. (E) NIH/3T3 knockout (KO) or vehicle cells were treated with (+) or without (-) 200 nM SAG. Gli1 was analyzed by qRT-PCR. Data are shown as the mean ± SEM (n=3). *P < 0.05, **P < 0.01, ***P < 0.001, ****P < 0.0001. All experiments were repeated three times independently.


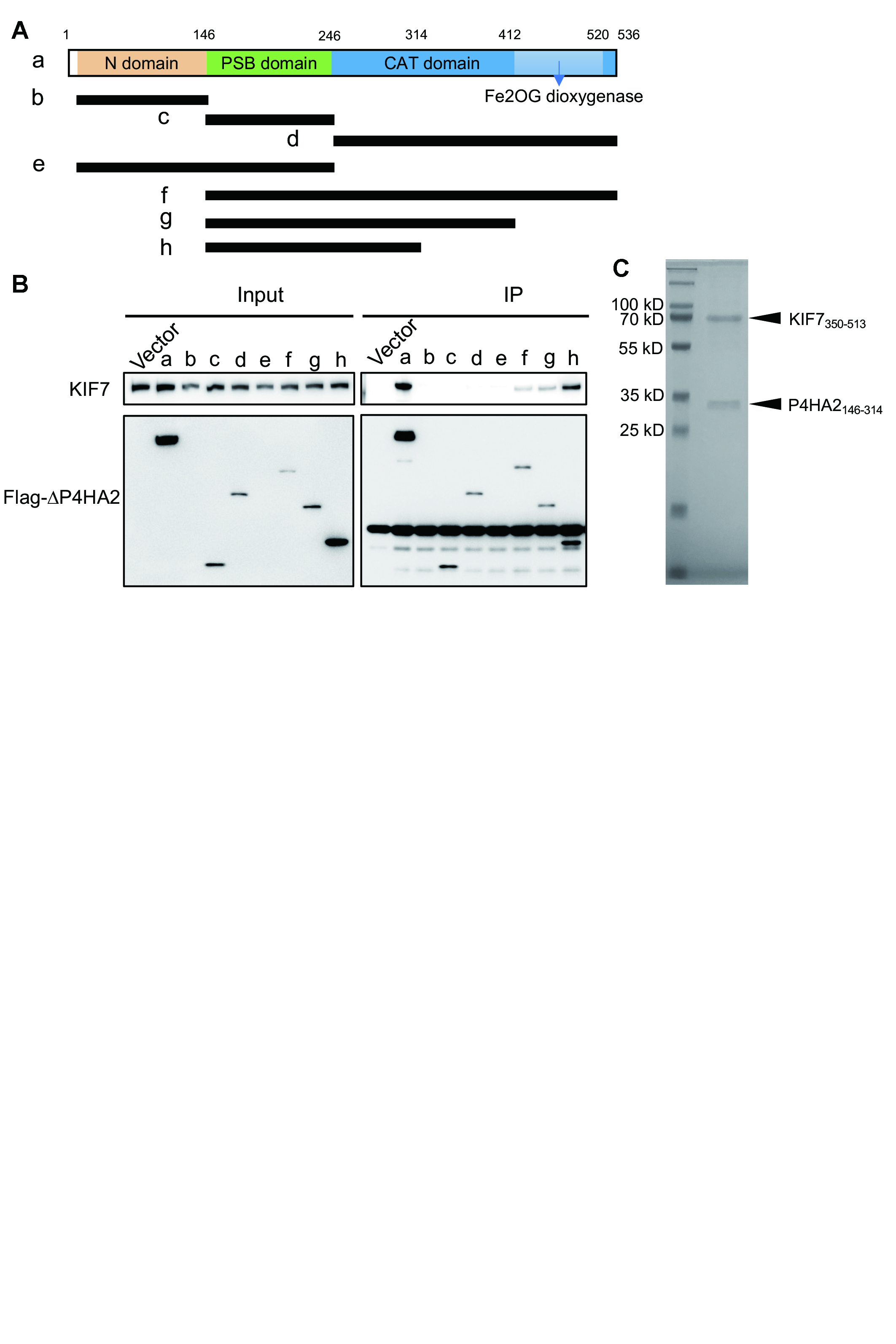


**Supplemental** **Figure 5. Mapping the interaction domains of KIF7 and P4HA2.** (A) Schematic of full-length P4HA2 proteins. The P4HA2 protein encodes a 536 aa protein with a N domain, peptide-substrate-binding domain (PSB domain) and a catalytic domain (CAT domain) representing the Fe2OG dioxygenase activity. Various truncated mutation constructs of P4HA2-Flag are shown schematically. (B) Mapping the interaction domain of P4HA2 with KIF7. HEK293T cells were transfected with constructs shown as (A). Cell lysates were prepared and subjected to immunoprecipitation (IP) with M2 anti-Flag beads. (C) His-tagged KIF7_350-513_ and Flag-tagged P4HA2_146-314_ proteins were purified and incubated. After His pulldown, the complex was defined.


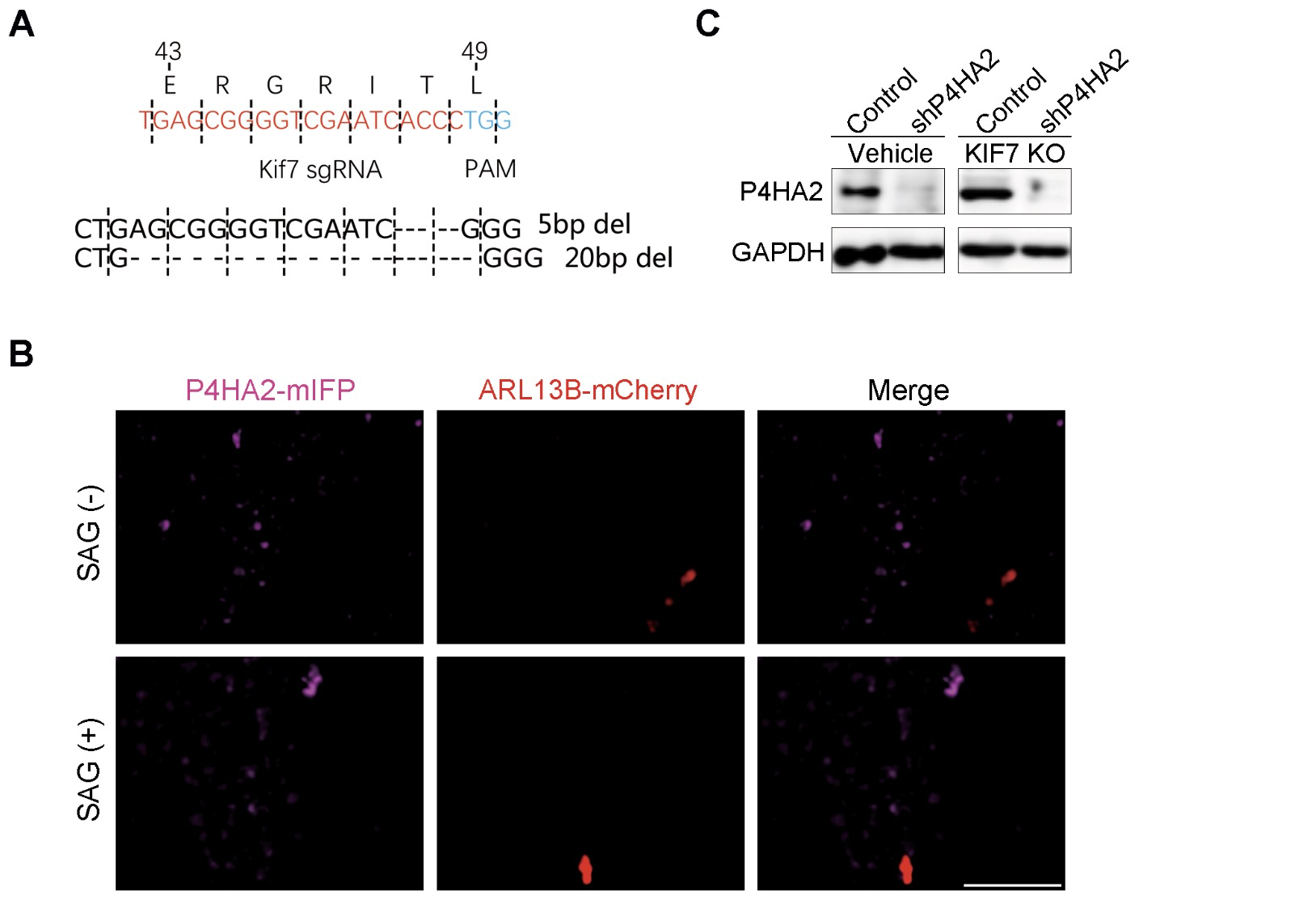


**Supplemental** **Figure 6. P4HA2 translocates with KIF7 in response to the Hh signals.** (A) Sequencing validation of KIF7 knockout in NIH/3T3 cells. Genomic DNA was amplified by PCR and sequenced. (B) NIH/3T3 control or KIF7 KO cells were co-infected with ARL13B-mCherry and P4HA2-mIFP lenti-virus. Cells were treated with 200 nM SAG (+) or not (-). mIFP (purple) to mark P4HA2, mCherry (red) to mark cilia, Scale bars: 5 μm. (C) NIH/3T3 KIF7 KO or vehicle cells were infected with shP4HA2 and the control lenti-virus, respectively.


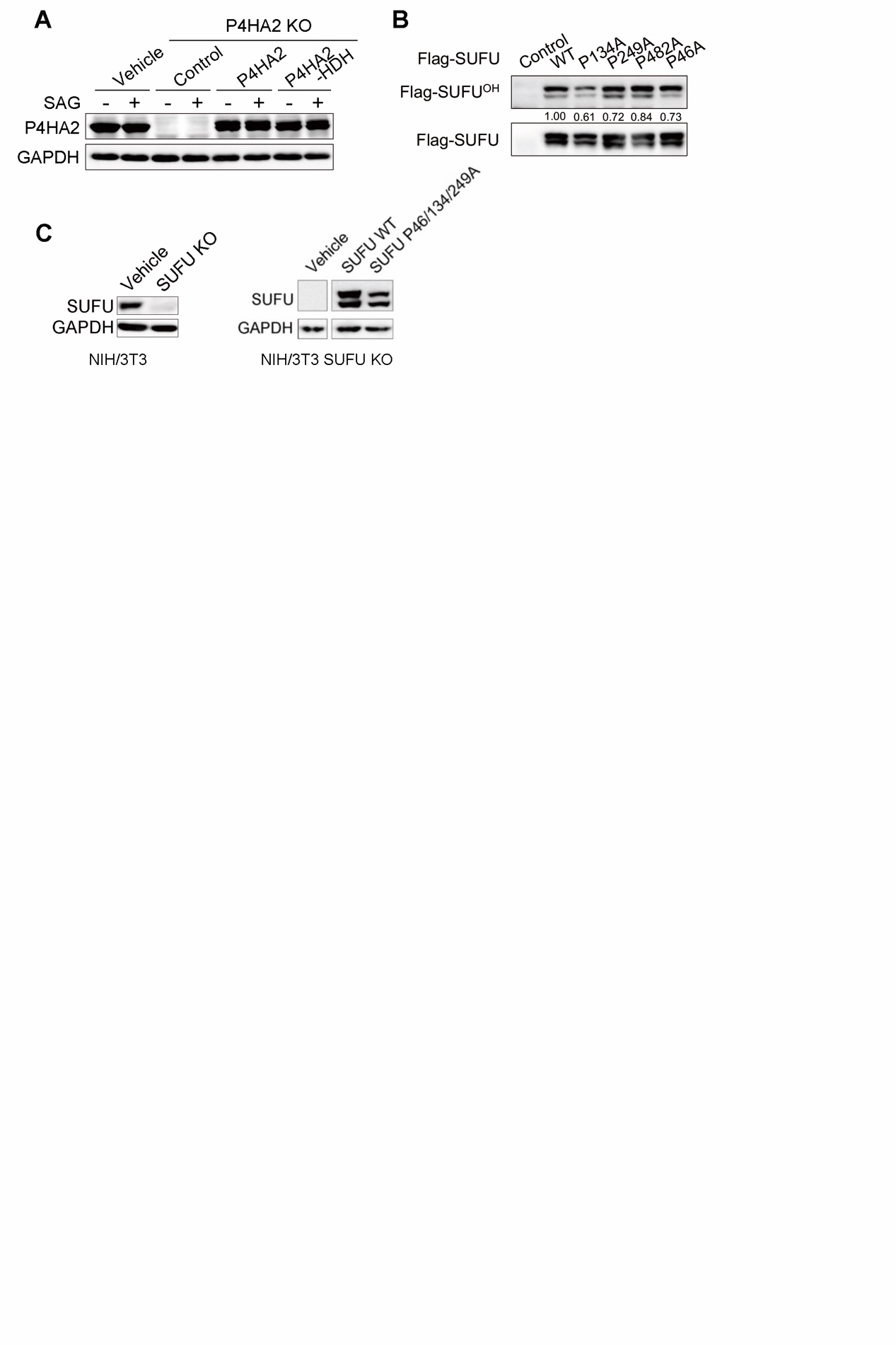


**Supplemental** **Figure 7. The regulation of P4HA2 on the Hh signaling dependents on its hydroxylase activity.** (A) Expression of P4HA2 or P4HA2-HDH in P4HA2 KO cells. (B) The 46^th^, 134^th^, 249^th^ and 482^th^ proline used site-directed mutagenesis (proline to alanine, P-A) and hydroxylated modifications was analyzed and normalized. (C) SUFU knockout NIH/3T3 cell line was constructed. Verification of SUFU knockout and rescue expression.


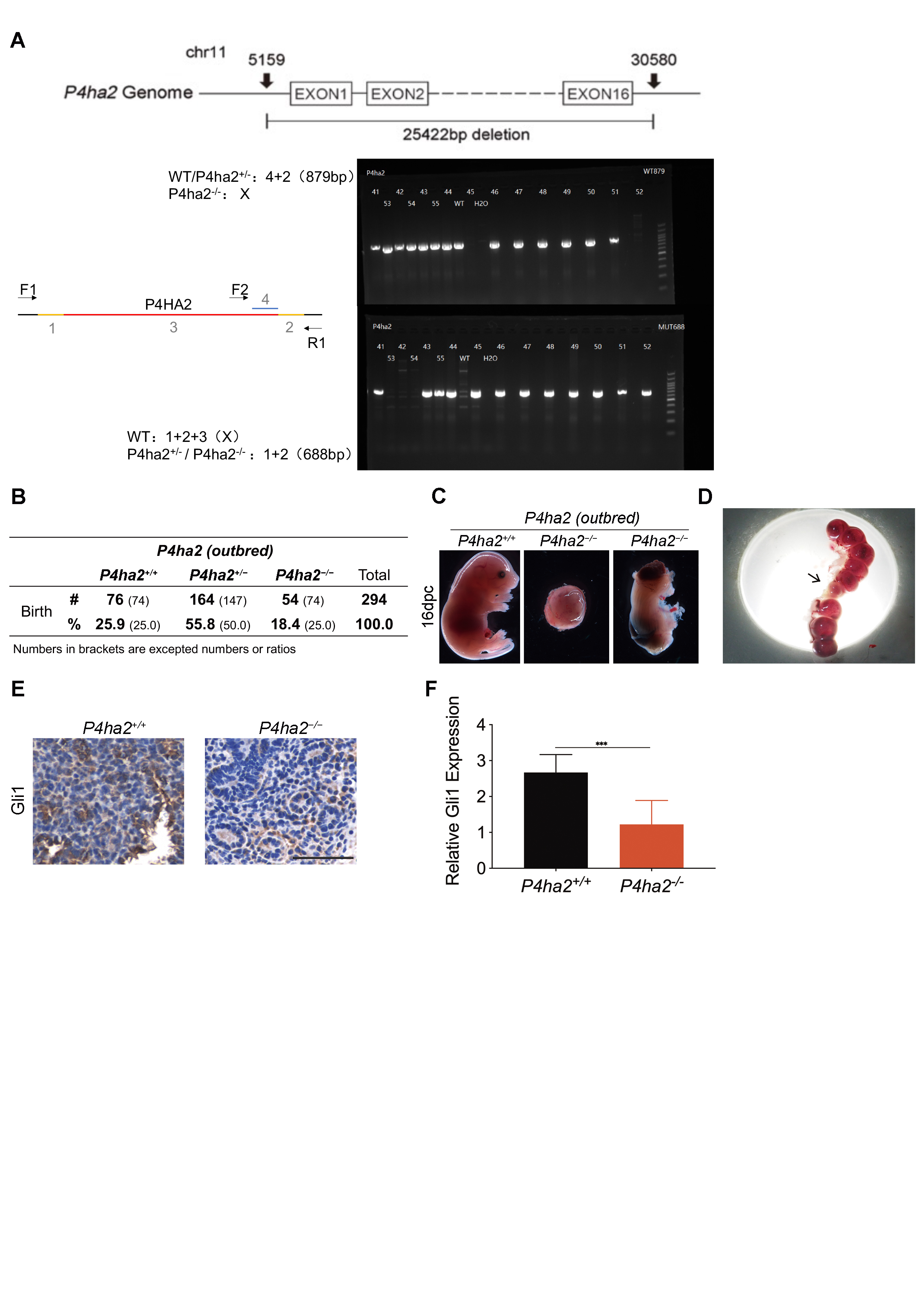


**Supplemental** **Figure 8. P4HA2 regulates the Hh pathway transduction *in vivo*.** (A) The construction of *P4ha2* knockout mice. (B) The birth of *P4ha2* knockout homozygous (*P4ha2^−/−^*) mice decreased. Breeding summary of *P4ha2* knockout heterozygote (*P4ha2^+/−^*) mouse intercrosses at birth and during embryogenesis. Numbers and percentages in brackets are expected numbers and ratios. *P4ha2^+/+^*, *P4ha2* wild-type littermate mice. (C) Complete absence of P4ha2 in mice leads to partial embryonic lethality. Representative images of the 16 days post crush (dpc) *P4ha2^+/+^* and *P4ha2^−/−^* embryos. Scale bars: 0.5 cm. (D) The represent image of uterus showed that one of the homozygous *P4ha2^−/−^* mice died in uterus. The black arrow indicates the stillborn fetus. (E) The expression of Gli1 is lower in *P4ha2^−/−^* embryos. Representative images of the immunohistochemical analysis of Gli1 expression in the lungs of 16-dpc mice embryos. Scale bars: 100 μm. (F) Statistical analysis of the relative Gli1 expression in *P4ha2^+/+^* and *P4ha2^−/−^* embryos by qRT-PCR. Data are shown as the mean ± SEM (n=9). ***P < 0.001.


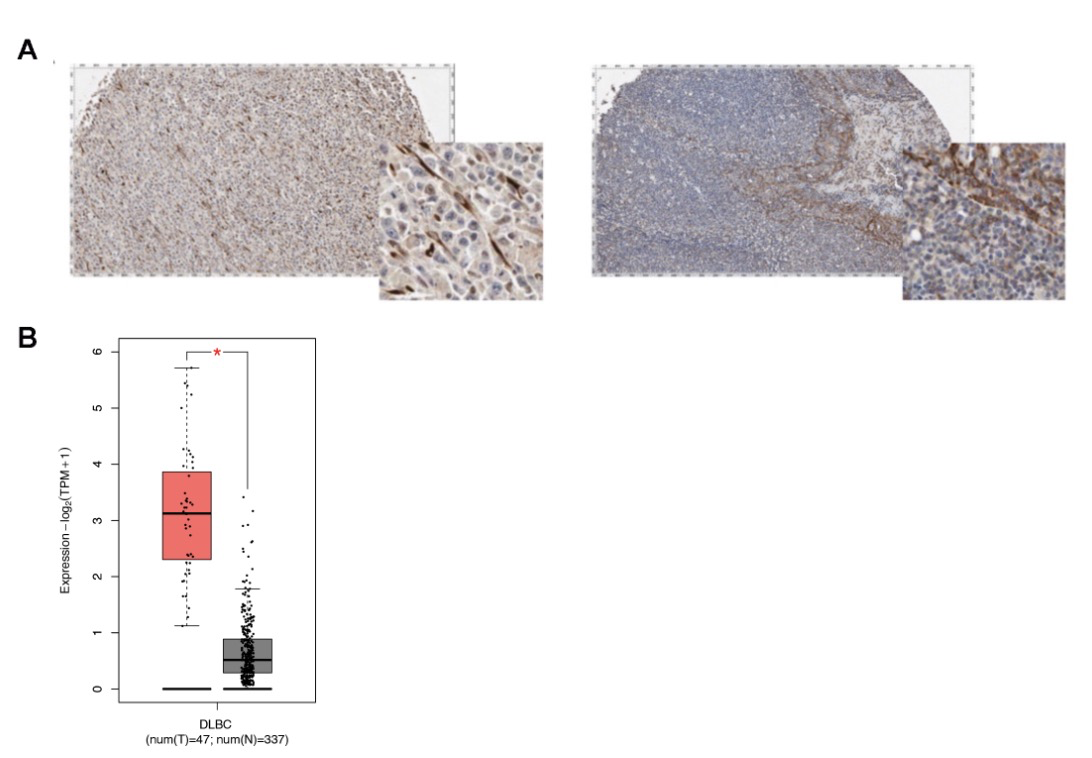


**Supplemental** **Figure 8. P4HA2 is highly expressed in DLBCL samples.** (A) P4HA2 is highly expressed in tumor stromal fibroblasts in DLBCL samples. The patient samples were analyzed from *Jiang, Blood, 2018.* (B) P4HA2 is highly expressed in DLBCL patients from TCGA database.


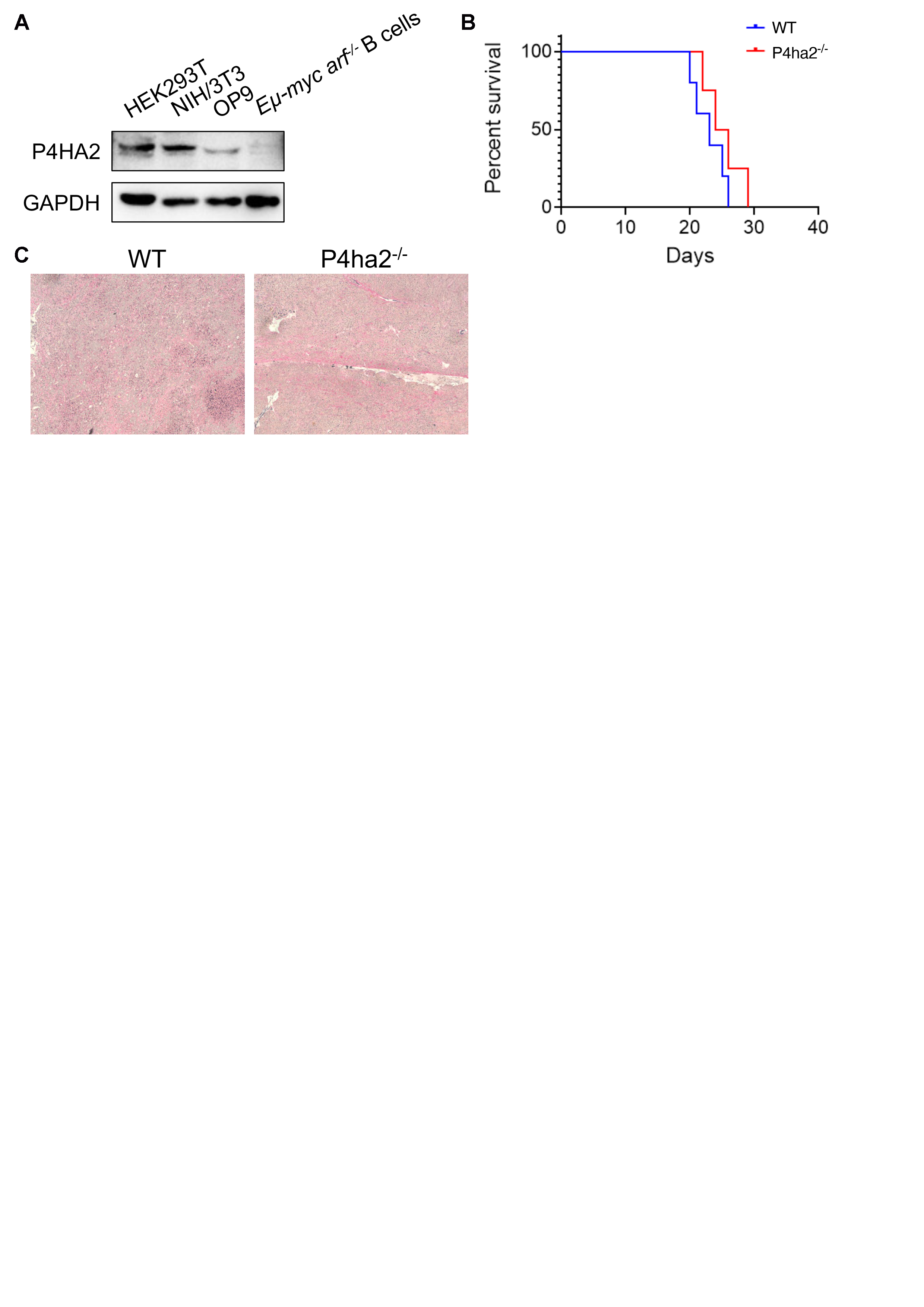


**Supplemental** **Figure 10. The tumor progression is impeded in *P4ha2* knockout mice.** (A) P4HA2 hardly expresses in *Eµ-myc arf^-/-^* B cells. (B) The overall survival of tumor-bearing mice. (WT mice : *P4ha2^-/-^* mice=5:4) (C) Staining collagen with Sirius Red, collagen levels are almost no difference between WT and *P4ha2^-/-^* mice xenograft.


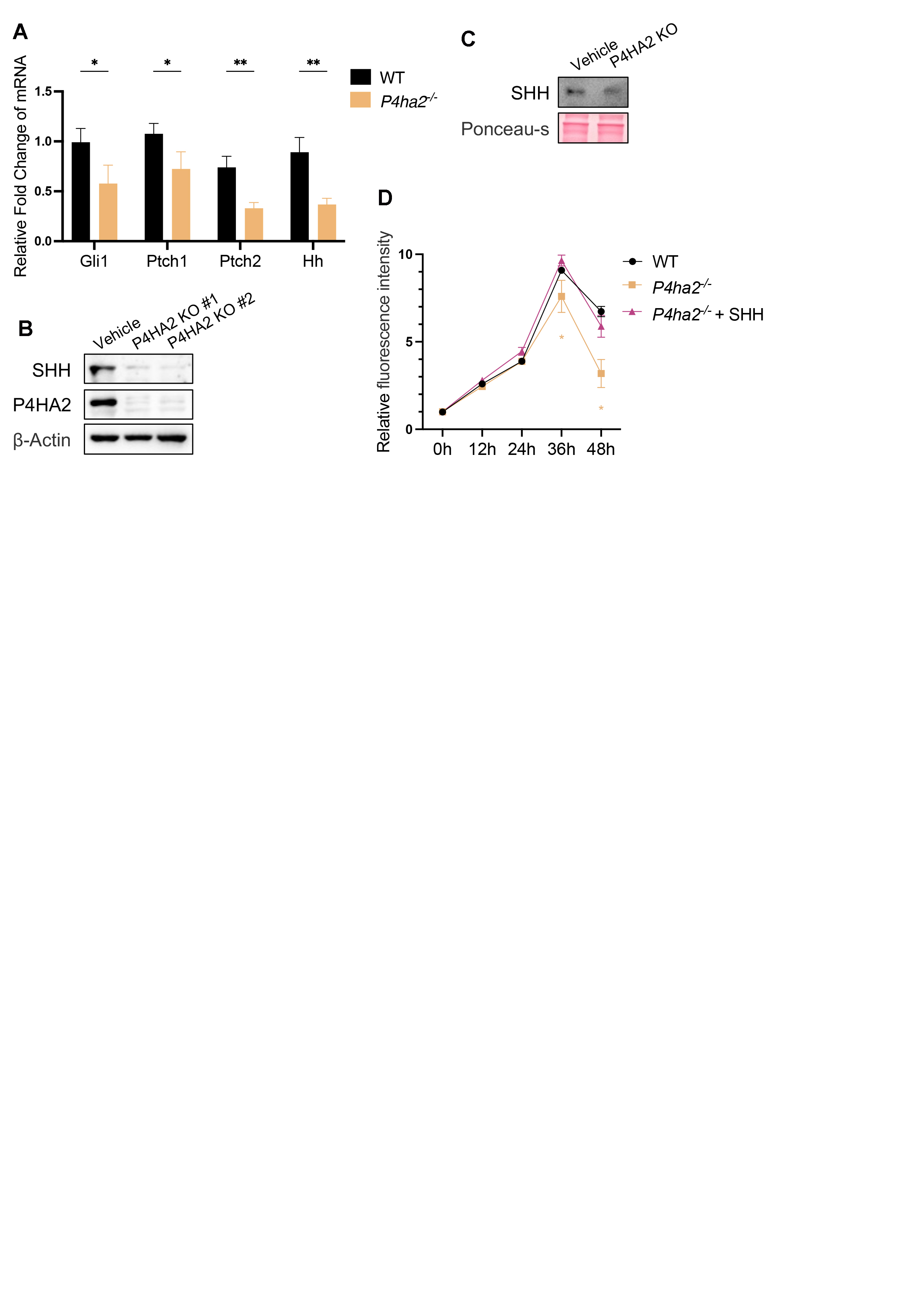


**Supplemental** **Figure 11. SHH is downregulated in P4HA2 knockout stromal cells.** (A) Primary stromal fibroblasts were isolated from spleen of C57BL/6 WT and *P4ha2*^-/-^ mice. The expression of Gli1, Ptch1, Ptch2 and HH ligands was detected by qRT-PCR from the primary stromal fibroblasts. (B) SHH expression was detected in OP9 cell lysates. (C) Secreted SHH was detected in OP9 cultured supernatant. (D) The growth curve of *Eµ-myc arf^-/-^* B cells co-cultured with primary bone marrow stromal fibroblasts supernatant. WT and *P4ha2*^-/-^ primary bone marrow fibroblasts were isolated and cultured. After cell counting, both primary cells were cultured at the same density, and the cultured supernatant was collected for co-culture with *Eµ-myc arf^-/-^* B cells. SHH was added to rescue the growth of *Eµ-myc arf^-/-^* B cells. The cell viability was detected every 12 hr.

**
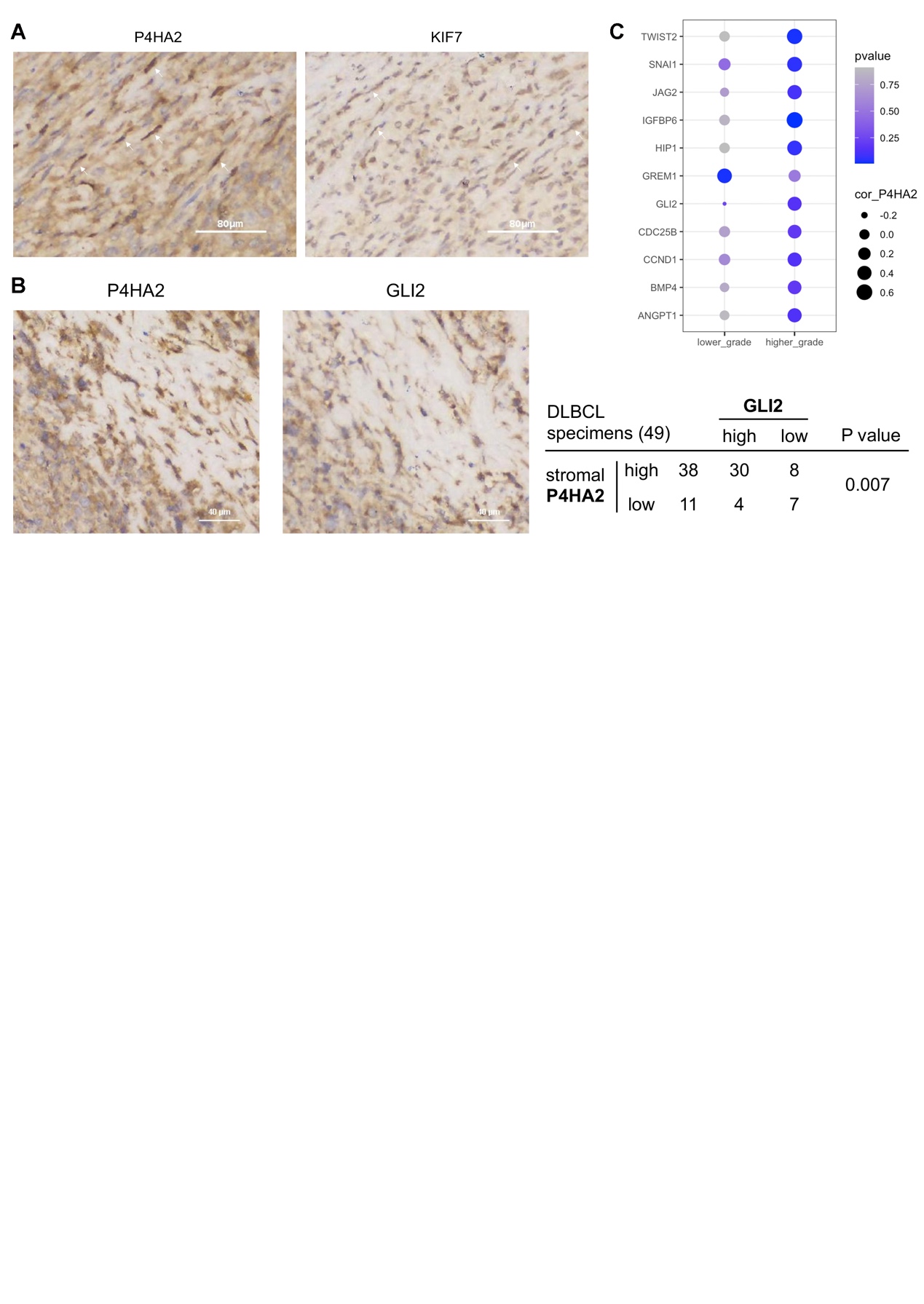
**

**Supplemental** **Figure 12. P4HA2 expression is correlated with the Hh pathway in DLBCL specimens.** (A) IHC staining of P4HA2 and KIF7 in human DLBCL pathological serial section. White arrows indicate the expression of P4HA2 and KIF7 in stromal fibroblasts. Scale bars: 80 μm. (B) Correlation between the expression of P4HA2 and GLI2 in human DLBCL tissues. Representative IHC staining of stromal P4HA2 and GLI2 in consecutive sections of DLBCL specimens (left panel). Scale bars: 40 μm. The relationship between P4HA2 and GLI2 staining status (right panel). Statistical significance was determined by χ2-test. (C) Spearman correlation between P4HA2 and Hedgehog downstream genes in high and low grades in the TCGA database. The size and color of each circle represent the relative correlation and p value (spearman), respectively. cor_P4HA2: Spearman correlation; lower_grade: I, II stages; higher_grade: III, IV stages.


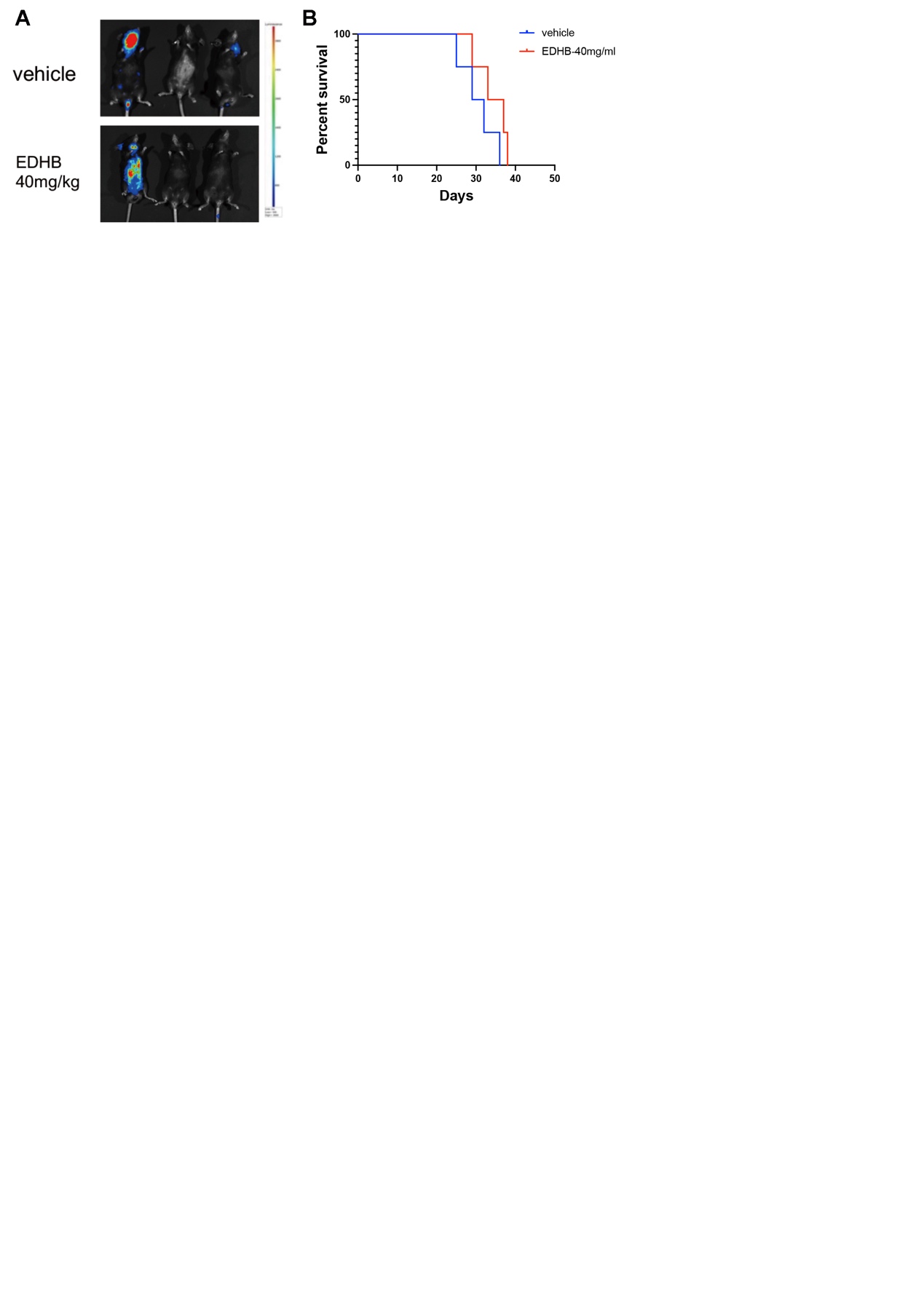


**Supplemental** **Figure 13. C-P4H inhibitor EDHB possesses anti-tumor effects.** (A) Bioluminescence imaging of C57BL/6 WT mice for 18 days after injection of luciferased *Eµ-myc arf^-/-^* B cells. (B) The overall survival of tumor-bearing mice. (Vehicle : EDHB = 4:4)

**Supplemental** **Table**

**Supplemental** **Table 1. Enriched proteins in P4HA2 precipitations.**

|  | | | |
| --- | --- | --- | --- |
| Software: | Mascot version 2.4.0 | |  |
| Database | Uniprot_human | |  |
| Enzyme | Trypsin |  |  |
| Maximum Missed Cleavages | 2 |  |  |
| Mass values | Monoisotopic | |  |
| Peptide Ions score cut-off | 20 |  |  |
| Instrument type | ESI-QUAD |  |  |
|  |  |  |  |
| 1. The proteins identified for a sample are listed in the order of high confidence to low confidence. The higher the protein score, the higher the protein identification confidence is. #N/A represents no data available. 2. Database search reference: Electrophoresis, 20(18) 3551-67 (1999). Its website is http://www.matrixscience.com. | | | |
| **Note**: "**prot_acc**": uniprot database unique identifier; "**gene_name**": gene name; "**prot_desc**": uniprot database protein description; "**prot_score**": protein score by mascot software; "**prot_mass**": protein mass (Dalton). | | | |
| **prot_acc** | **gene_name** | **prot_desc** | **prot_score** |
| O15460 | P4HA2 | Prolyl 4-hydroxylase subunit alpha-2 OS=Homo sapiens GN=P4HA2 PE=1 SV=1 | 1066 |
| Q2M1P5 | KIF7 | Kinesin-like protein KIF7 OS=Homo sapiens GN=KIF7 PE=1 SV=2 | 864 |
| Q13085 | ACACA | Acetyl-CoA carboxylase 1 OS=Homo sapiens GN=ACACA PE=1 SV=2 | 625 |
| P09874 | PARP1 | Poly [ADP-ribose] polymerase 1 OS=Homo sapiens GN=PARP1 PE=1 SV=4 | 451 |
| P11142 | HSPA8 | Heat shock cognate 71 kDa protein OS=Homo sapiens GN=HSPA8 PE=1 SV=1 | 445 |
| P60709 | ACTB | Actin, cytoplasmic 1 OS=Homo sapiens GN=ACTB PE=1 SV=1 | 437 |
| P08107 | HSPA1A | Heat shock 70 kDa protein 1A/1B OS=Homo sapiens GN=HSPA1A PE=1 SV=5 | 357 |
| P05141 | SLC25A5 | ADP/ATP translocase 2 OS=Homo sapiens GN=SLC25A5 PE=1 SV=7 | 313 |
| I3L2P8 | P4HB | Protein disulfide-isomerase OS=Homo sapiens GN=P4HB PE=2 SV=1 | 280 |
| F5GWP8 | JUP | Junction plakoglobin OS=Homo sapiens GN=JUP PE=2 SV=1 | 278 |
| P15924 | DSP | Desmoplakin OS=Homo sapiens GN=DSP PE=1 SV=3 | 267 |
| P11021 | HSPA5 | 78 kDa glucose-regulated protein OS=Homo sapiens GN=HSPA5 PE=1 SV=2 | 257 |
| Q5JP53 | TUBB | Tubulin beta chain OS=Homo sapiens GN=TUBB PE=3 SV=1 | 234 |
| P34931 | HSPA1L | Heat shock 70 kDa protein 1-like OS=Homo sapiens GN=HSPA1L PE=1 SV=2 | 216 |
| J3KN86 | RPS3 | 40S ribosomal protein S3 OS=Homo sapiens GN=RPS3 PE=3 SV=1 | 214 |
| H0Y3Z3 | P4HB | Protein disulfide-isomerase (Fragment) OS=Homo sapiens GN=P4HB PE=3 SV=1 | 211 |
| P17066 | HSPA6 | Heat shock 70 kDa protein 6 OS=Homo sapiens GN=HSPA6 PE=1 SV=2 | 207 |
| Q10570 | CPSF1 | Cleavage and polyadenylation specificity factor subunit 1 OS=Homo sapiens GN=CPSF1 PE=1 SV=2 | 206 |
| O43809 | NUDT21 | Cleavage and polyadenylation specificity factor subunit 5 OS=Homo sapiens GN=NUDT21 PE=1 SV=1 | 156 |
| Q562R1 | ACTBL2 | Beta-actin-like protein 2 OS=Homo sapiens GN=ACTBL2 PE=1 SV=2 | 155 |
| F8WJN3 | CPSF6 | Cleavage and polyadenylation-specificity factor subunit 6 OS=Homo sapiens GN=CPSF6 PE=2 SV=1 | 150 |
| P12236 | SLC25A6 | ADP/ATP translocase 3 OS=Homo sapiens GN=SLC25A6 PE=1 SV=4 | 143 |
| G8JLB6 | HNRNPH1 | Heterogeneous nuclear ribonucleoprotein H OS=Homo sapiens GN=HNRNPH1 PE=2 SV=1 | 141 |
| P38646 | HSPA9 | Stress-70 protein, mitochondrial OS=Homo sapiens GN=HSPA9 PE=1 SV=2 | 139 |
| P19338 | NCL | Nucleolin OS=Homo sapiens GN=NCL PE=1 SV=3 | 137 |
| P08238 | HSP90AB1 | Heat shock protein HSP 90-beta OS=Homo sapiens GN=HSP90AB1 PE=1 SV=4 | 130 |
| Q5VTE0 | EEF1A1P5 | Putative elongation factor 1-alpha-like 3 OS=Homo sapiens GN=EEF1A1P5 PE=5 SV=1 | 129 |
| P10412 | HIST1H1E | Histone H1.4 OS=Homo sapiens GN=HIST1H1E PE=1 SV=2 | 122 |
| H0YA55 | ALB | Serum albumin (Fragment) OS=Homo sapiens GN=ALB PE=4 SV=1 | 121 |
| J3KP72 | PCMT1 | Protein-L-isoaspartate O-methyltransferase OS=Homo sapiens GN=PCMT1 PE=3 SV=1 | 121 |
| P01857 | IGHG1 | Ig gamma-1 chain C region OS=Homo sapiens GN=IGHG1 PE=1 SV=1 | 120 |

**Supplemental** **Table 2. Primers for sgRNA, shRNA, qRT-PCR and Genotyping target sequence.**

| **sgRNA oligonucleotides** |
| --- |
| sgRNA mouse Negative Control: GCGAGGTATTCGGCTCCGCG |
| P4ha1 mouse sgRNA target sequence -1: TGACGTAAAAAACCCTGGAT |
| P4ha1 mouse sgRNA target sequence -2: TTGTTGCTGTACTCGTTAGC |
| P4ha2 mouse sgRNA target sequence -1: CCAGCTCAGGACACCGAACC |
| P4ha2 mouse sgRNA target sequence -2: CGATCTGATTTACGCAGAGA |
| Sufu mouse sgRNA target sequence -1: GCGGCGACACTCTCCGTAGA |
| Sufu mouse sgRNA target sequence -2: ACTTGACGATAGCGGTAACC |
| Kif7 mouse sgRNA target sequence -1: AGGGTGATTCGACCCCGCTC |
| Kif7 mouse sgRNA target sequence -2: TGAGCGGGGTCGAATCACCC |
| Mice KO gRNA target sequence-1 (matching reverse strand of gene): ATGAAGGGTGACGTATCAGTGGG |
| Mice KO gRNA target sequence-2 (matching reverse strand of gene): CCGTGCCGGGTGCTATAGTCTGG |
| sgRNA Human Negative Control: ACGGAGGCTAAGCGTCGCAA |
| P4HA2 Human sgRNA target sequence -1: CCGAATTCTTCACCTCTATT |
| P4HA2 Human sgRNA target sequence -2: ACAGCTCAGGACACCAAACC |
| **shRNA oligonucleotides** |
| shRNA Negative Control: TTCTCCGAACGTGTCACGT |
| P4ha2 mouse shRNA target sequence -1: CCAAATCATCTTCAAGTTCAA |
| P4ha2 mouse shRNA target sequence -2: GATGGAGCAGGTACTGAAG |
| P4ha1 mouse hRNA target sequence -1: GACATCACTAAAAGATTAT |
| P4ha1 mouse shRNA target sequence -2: ATGGTAACTTAGTATATTT |
| **qRT-PCR oligonucleotides** |
| qRT-PCR human GAPDH Forward sequence: CTTTGGTATCGTGGAAGGACTC |
| qRT-PCR human GAPDH Reverse sequence: GTAGAGGCAGGGATGATGTTC |
| qRT-PCR human GLI1 Forward sequence: AATGCTGCCATGGATGCTAGA |
| qRT-PCR human GLI1 Reverse sequence: GAGTATCAGTAGGTGGGAAGTCCATAT |
| qRT-PCR human GLI2 Forward sequence: CACCAAGAGATACACAGACCC |
| qRT-PCR human GLI2 Reverse sequence: TCACTGTCCCCATTCTCTTTG |
| qRT-PCR human PTCH1 Forward sequence: GGGTGGCACAGTCAAGAACAG |
| qRT-PCR human PTCH1 Reverse sequence: TACCCCTTGAAGTGCTCGTACA |
| qRT-PCR human PTCH2 Forward sequence: TCACACCCGAAGCACTTGG |
| qRT-PCR human PTCH2Reverse sequence: ATCATCCGCTCAATCATTCCATT |
| qRT-PCR mouse Gapdh Forward sequence: TGTGTCCGTCGTGGATCTGA |
| qRT-PCR mouse Gapdh Reverse sequence: CCTGCTTCACCACCTTCTTGAT |
| qRT-PCR mouse Gli1 Forward sequence: CCAAGCCAACTTTATGTCAGGG |
| qRT-PCR mouse Gli1 Reverse sequence: AGCCCGCTTCTTTGTTAATTTGA |
| qRT-PCR mouse Gli2 Forward sequence: TTTGCCGATTGACATGAGACA |
| qRT-PCR mouse Gli2 Reverse sequence: GGTGGGAGGCCCGTGTAC |
| qRT-PCR mouse Ptch1 Forward sequence: CCCTAACAAAAATTCAACCAAACCT |
| qRT-PCR mouse Ptch1 Reverse sequence: GCATATACTTCCTGGATAAACCTTGAC |
| qRT-PCR mouse Ptch2 Forward sequence: ACAGCTGGCCTCATAGTGCT |
| qRT-PCR mouse Ptch2 Reverse sequence: GGGATGGCACTCAGTTTGAT |
| qRT-PCR mouse Shh Forward sequence: TCCAAAGCTCACATCCACTG |
| qRT-PCR mouse Shh Reverse sequence: CGTAAGTCCTTCACCAGCTTG |
| qRT-PCR mouse Ihh Forward sequence: CCCAACTACAATCCCGACATC |
| qRT-PCR mouse Ihh Reverse sequence: TCACCCGCAGTTTCACAC |
| qRT-PCR mouse Dhh Forward sequence: ATCCACGTATCGGTCAAAGC |
| qRT-PCR mouse Dhh Reverse sequence: CACGATGTAGTTCCCTCAGC |
| **Genotyping oligonucleotides** |
| Mice Genotyping PCR Primers Forward sequence1: GGAGAGAAGAATGGGAAACAACCAG |
| Mice Genotyping PCR Primers Reverse sequence 1: GAGATTATAGGGAGGCACTGTCAG |
| Mice Genotyping PCR Primers Forward sequence 2: GCTGCTTGCTGAACTGATAGGTG |
